# Artificial Intelligence for Colorectal Cancer Diagnosis: A Comprehensive Survey of Modalities, Frameworks, and Clinical Tasks

**DOI:** 10.3390/cancers18213522

**Published:** 2026-11-02

**Authors:** Bassant El-Mosalamy, Hossam Magdy Balaha, Sohayla El-Moursi, Dibson Gondim, Khaldon Lweesy, Jawad Yousaf, Mohammed Ghazal, Ayman El-Baz

**Affiliations:** 1 Department of Bioengineering, J.B. Speed School of Engineering, University of Louisville, Louisville, KY 40292, USA; bassant.elmosalamy@louisville.edu (B.E.-M.); hmbala01@louisville.edu (H.M.B.); sohayla.elmoursi.1@louisville.edu (S.E.-M.); 2 Department of Pathology and Laboratory Medicine, School of Medicine, University of Louisville, Louisville, KY 40292, USA; dibson.gondim@louisville.edu; 3 Electrical, Computer, and Biomedical Engineering Department, College of Engineering, Abu Dhabi University, Abu Dhabi 59911, United Arab Emirates; khaldon.lweesy@adu.ac.ae (K.L.); jawad.yousaf@adu.ac.ae (J.Y.); mohammed.ghazal@adu.ac.ae (M.G.)

**Keywords:** artificial intelligence, colonoscopy, colorectal cancer, computational pathology, computer-aided diagnosis, medical imaging, radiogenomics

## Abstract

The diagnosis of colorectal cancer encompasses nine complementary modalities: stool-based screening, blood-based biomarkers, cross-sectional imaging, endoscopic evaluation, histopathology, liquid biopsy, molecular and genetic profiling, microbiome analysis, and nanotechnology-based diagnostics. This narrative review synthesizes 160 references spanning seven task-specific artificial intelligence domains, including computer-aided detection, optical diagnosis, invasion depth prediction, computational pathology and radiogenomics, metastasis forecasting, neoadjuvant treatment response prediction, and surgical workflow analysis. We propose a conceptual multimodal pipeline comprising data ingestion, preprocessing and harmonization, feature representation, multimodal fusion, forecasting, explainability, and validation. A structured evidence grading framework classifies each clinical task by evidence level, consistency, and clinical readiness. We further catalog public datasets, challenge benchmarks, and five reporting standards: TRIPOD-AI, CONSORT-AI, SPIRIT-AI, DECIDE-AI, and CLAIM. Computer-aided detection and optical diagnosis demonstrate the highest maturity with Level 1 evidence from randomized controlled trials, while computational pathology, radiogenomics, and metastasis prediction remain at Level 2 with retrospective validation (internal or external). Microbiome screening, nanotechnology diagnostics, and surgical phase recognition are exploratory. The principal barriers to clinical adoption are domain shift, data scarcity, calibration, prospective validation, and workflow integration. We conclude that clinically useful artificial intelligence requires robust external validation, uncertainty quantification, explainable outputs, standardized reporting, and a structured translational pathway from retrospective evidence through regulatory clearance to post-deployment monitoring.

## Simple Summary

Colorectal cancer is a leading cause of cancer-related deaths globally, making early and accurate diagnosis essential. The diagnostic journey involves multiple modalities, including stool tests, cross-sectional imaging, endoscopy, and tissue pathology. Artificial intelligence (AI) is increasingly being integrated into these steps to improve diagnostic accuracy and clinical efficiency. This comprehensive review synthesizes findings from 160 studies to evaluate the current state of AI across nine diagnostic modalities and seven specific clinical tasks. Our analysis reveals that AI tools for real-time polyp detection and optical diagnosis during colonoscopies are the most clinically mature, supported by strong evidence from randomized controlled trials. In contrast, emerging applications such as predicting tumor genetics from pathology slides or forecasting metastasis from imaging remain in the retrospective validation phase. We also outline a conceptual multimodal AI pipeline and identify key barriers to clinical adoption, including domain shift, data scarcity, and the need for standardized reporting frameworks. That said, this review highlights that while AI holds immense potential to transform colorectal cancer diagnostics, robust external validation, uncertainty quantification, explainability, and seamless integration into clinical workflows are critical prerequisites for routine patient care.

## 1. Introduction

Colorectal cancer is a common malignancy and a leading cause of cancer death, accounting for approximately 1.9 million new cases and 900,000 deaths globally each year. Screening can detect precancerous lesions and localized tumors before the onset of symptoms, so it is beneficial for early disease detection [1,2]. Clinical diagnosis follows a multimodal pathway. Stool-based tests identify those at risk, blood-based biomarkers assist in surveillance, endoscopy provides direct visualization and tissue biopsy, imaging evaluates local and distant disease, histopathology confirms malignancy, and molecular profiling informs prognosis and therapy [3,4].

Artificial intelligence has become relevant at every step in this pathway. In endoscopy, computer-aided detection and diagnosis systems support the detection of polyps and optical biopsy [5,6,7]. In pathology, deep learning and weakly supervised frameworks permit tumor detection, tissue classification and molecular inference from whole-slide images [8,9,10]. In radiology, radiomics and deep learning are applied to disease staging, metastasis detection and prognostic predictions [11,12,13]. Research on phase recognition and human–artificial intelligence interaction in surgical workflows studies the functioning of models in clinical settings [14,15,16].

Despite this progress, translation remains challenging. Many studies rely on single-center data, disparate end points, and limited external validation. Calibration, uncertainty estimation, explainability and standardized reporting are also necessary for clinical deployment [17,18]. Therefore, this review integrates the diagnostic modalities, artificial intelligence frameworks, task-specific evidence, datasets and evaluation standards for the diagnosis of colorectal disease.

The remainder of this paper is organized as follows. Section 2 describes the review methodology and scope. Section 3 reviews the colorectal diagnosis modalities. Section 4 outlines the artificial intelligence frameworks. Section 5 synthesizes the task-specific studies. Section 6 catalogs the datasets, benchmarks, and evaluation standards. Section 7 discusses the open challenges and future directions, and Section 8 concludes the review.

## 2. Review Methodology and Scope

**Figure 1:**
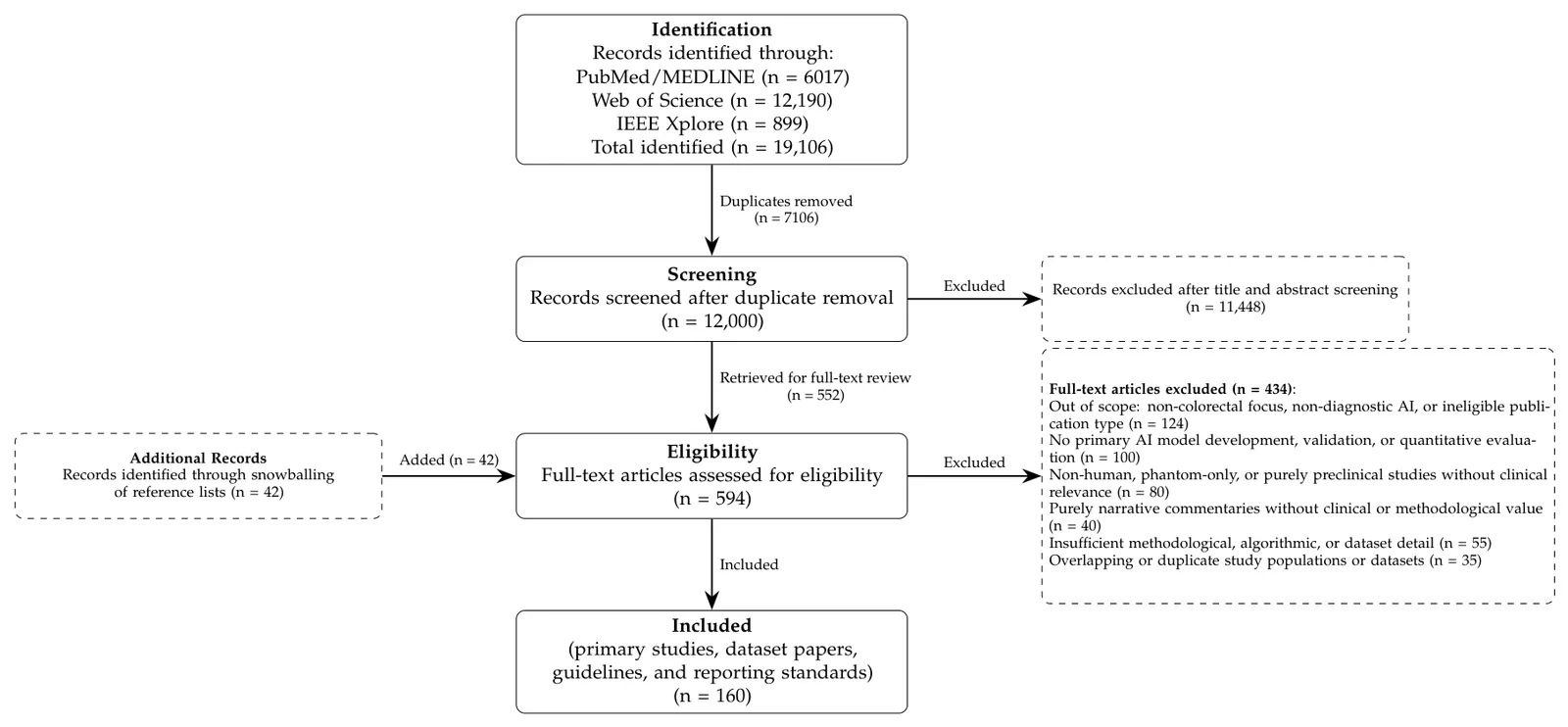
PRISMA 2020-style study selection flow diagram. The search and screening process yielded 160 included references, encompassing primary artificial intelligence studies, benchmark and dataset papers, clinical guidelines, and reporting standards. The 160 figure reflects the eligibility-screen survivors; secondary methodological and regulatory citations that appear in the reference list for context (e.g., PRISMA 2020 statement and FDA/EU MDR guidance) are not counted in the PRISMA flow.

This study is designed as a comprehensive narrative review that employed a systematic PRISMA-based literature search and selection process for primary studies supplemented by targeted inclusion for secondary studies [19]. While PRISMA is strictly designed for primary research, clinical guidelines, reporting standards, and benchmark datasets were integrated via snowballing to ensure comprehensive coverage. The search and selection procedure is outlined in Figure 1. The first search yielded 19,106 records. We removed 7106 duplicates and excluded 11,448 of these (a 95.4% exclusion rate justified by an intentionally broad initial search strategy to capture all the relevant colorectal AI research). The remaining 552 articles from the database search, along with 42 records identified through snowballing (totaling 594 full-text articles), were screened for eligibility. Of these, 434 articles were excluded because they did not meet the technical, clinical, or methodological criteria of the review. The final corpus consisted of 160 included references. This count reflects only primary studies, benchmark and dataset papers, clinical guidelines, and reporting standards that survived the full-text eligibility screen; it does not include secondary citations (e.g., methodological references such as the PRISMA 2020 statement [19] or regulatory documents [20,21,22,23,24]) that are cited in the bibliography for context but were not counted in the PRISMA flow. The last search date for each database was: PubMed/MEDLINE 28 August 2026; Web of Science 28 August 2026; IEEE Xplore 28 August 2026; Google Scholar was queried as a supplementary source on 28 August 2026 and is not counted as a primary database. The August 2026 limit reported in Appendix A is a publication-date cutoff applied to the Boolean search strings, not a search-execution date.

### 2.1. Search Strategy and Sources

The search combined colorectal disease terms with artificial intelligence terms (full search strings, dates, and limits are provided in Appendix A). No formal protocol was registered in PROSPERO; however, the review was conducted in strict accordance with the PRISMA 2020 guidelines. The representative disease terms included colorectal cancer, colon cancer, rectal cancer, polyp, colonoscopy, CT colonography, histopathology, and biopsy. The representative artificial intelligence terms included machine learning, deep learning, convolutional neural network, transformer, radiomics, computational pathology, computer-aided detection, computer-aided diagnosis, and surgical data science. Bibliographies of included reviews were screened for additional relevant studies.

### 2.2. Study Selection and Data Extraction

Two reviewers screened records independently at the title and abstract screening stage, achieving a Cohen’s kappa of 0.88. Disagreements were resolved by consensus. For included studies, reviewers extracted the data type, model architecture, clinical task, validation strategy, performance metrics, and principal limitations. When available, external validation, prospective evaluation, and human–artificial intelligence interaction results were prioritized. Refer to Table 1.

### 2.3. Scope

The review focuses on diagnosis and closely related prognostic tasks. Detection, characterization, invasion depth prediction, neoadjuvant treatment response prediction, molecular phenotype inference, metastasis forecasting, surgical phase recognition, and human–artificial intelligence interaction are included. Pure treatment selection, drug development, and epidemiological modeling without diagnostic data are outside the primary scope.

### 2.4. Evidence Grading

Because included studies report heterogeneous metrics, validation designs, and population characteristics, formal meta-analysis was not feasible for the overall synthesis. Instead, a structured qualitative evidence grading and narrative synthesis were applied to each task-specific subsection in Section 5. The first two authors independently assigned the evidence grades, and any discrepancies were resolved by consensus with the senior author. Each task was graded on three axes (Refer to Table 2):

1. Evidence level: Level 1: at least two prospective or randomized studies with external validation. Level 2: multiple retrospective studies with internal or external validation. Level 3: single retrospective studies, case series, or preclinical work.
2. Consistency: High: direction and magnitude of effect agree across studies. Moderate: some disagreement in magnitude. Low: conflicting results or insufficient studies.
3. Clinical readiness: Deployed: integrated into clinical workflow with regulatory clearance. Validated: prospective or multi-site validation completed but not yet deployed. Exploratory: retrospective or single-site evidence only.

### 2.5. Comparison with Existing Surveys

Several prior reviews have examined artificial intelligence in colorectal disease; however, three critical gaps persist in the existing literature. First, the prior surveys predominantly focus on a single modality or a narrow task spectrum, leaving the cross-modality diagnostic pipeline fragmented. Second, task-specific evidence has not been systematically linked to reporting governance frameworks, making it difficult for readers to assess the translational readiness of reported models. Third, emerging diagnostic modalities such as nanotechnology-based biosensors, microbiome profiling, and volatile organic compound screening have been largely omitted from prior artificial intelligence surveys. Table 3 contrasts the present survey with four recent reviews: Liang et al. [25], who focused on endoscopic deep learning; Reitsam et al. [10], who surveyed computational pathology; Spinelli et al. [26], who reviewed surgical artificial intelligence; and Nazarian et al. [27], who examined endoscopic computer-aided diagnosis. While prior studies, such as Mehta et al. [28], have validated the clinical effectiveness of specific artificial intelligence systems, no existing survey integrates all nine diagnostic modality categories within a unified multimodal pipeline framework. The present work addresses these gaps by (i) covering the full diagnostic spectrum from stool-based screening to nanotechnology, (ii) coupling task-specific evidence with structured evidence grading and reporting standards (TRIPOD-AI, CONSORT-AI, SPIRIT-AI, DECIDE-AI, and CLAIM), and (iii) providing a comprehensive dataset and benchmark catalog alongside translational barrier analysis.

## 3. Colorectal Diagnosis Modalities

**Figure 2:**
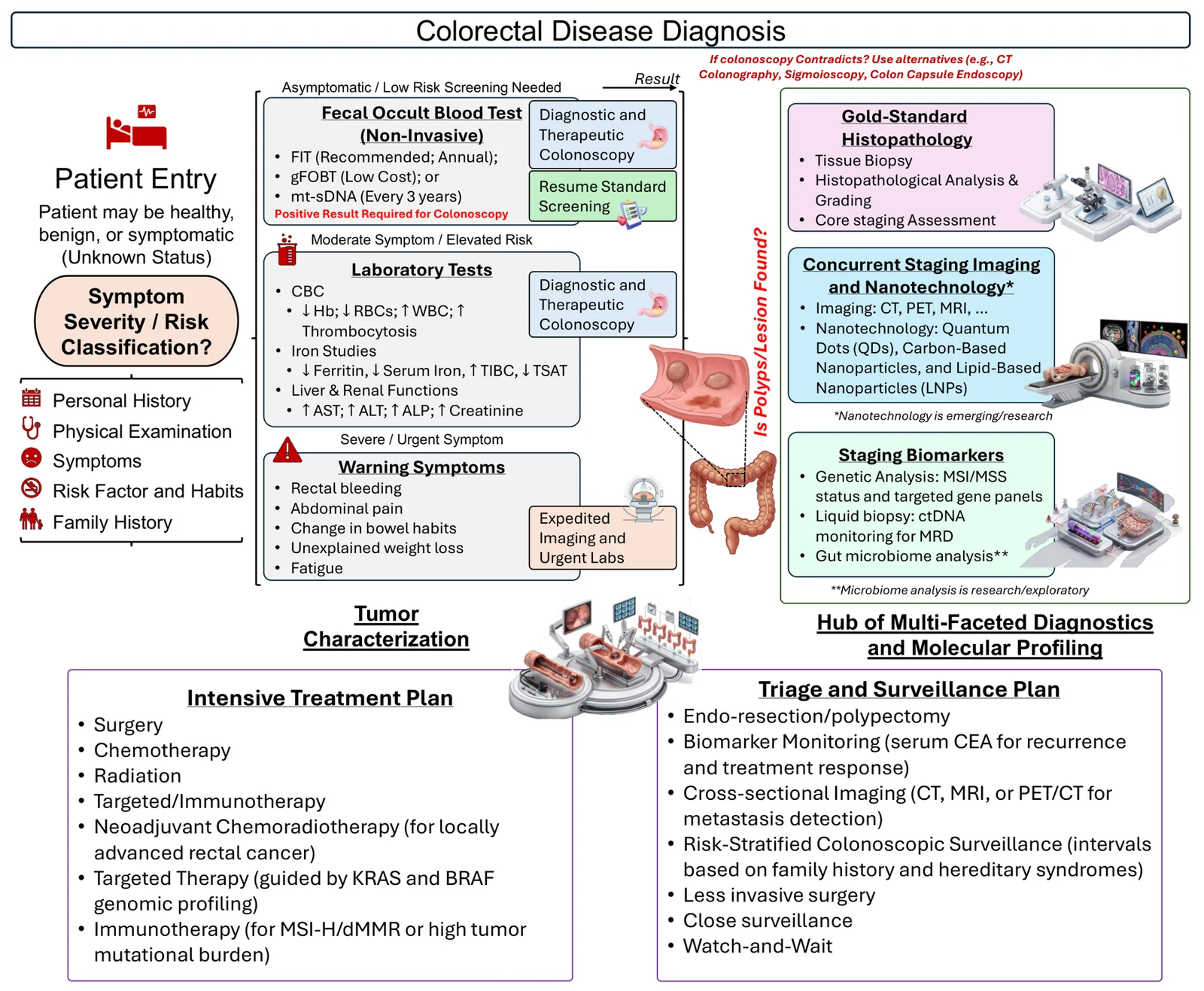
Comprehensive clinical diagnostic and treatment pathway for colorectal disease. The workflow begins with patient entry and risk stratification based on symptom severity and clinical history. Asymptomatic or low-risk individuals undergo non-invasive fecal occult blood testing, whereas symptomatic patients receive laboratory tests and expedited imaging. Positive screening results or elevated risk indications prompt diagnostic and therapeutic colonoscopy. Upon lesion detection, tumor characterization is performed via a multimodal hub encompassing gold-standard histopathology, concurrent staging imaging, emerging nanotechnology platforms, and molecular staging biomarkers. Based on the comprehensive diagnostic and staging outcomes, patients are directed into either an Intensive Treatment Plan (incorporating surgery, chemotherapy, radiation, and targeted or immunotherapy) or a Triage and Surveillance Plan (encompassing endo-resection, biomarker monitoring, cross-sectional imaging, and watch-and-wait strategies).

**Figure 3:**
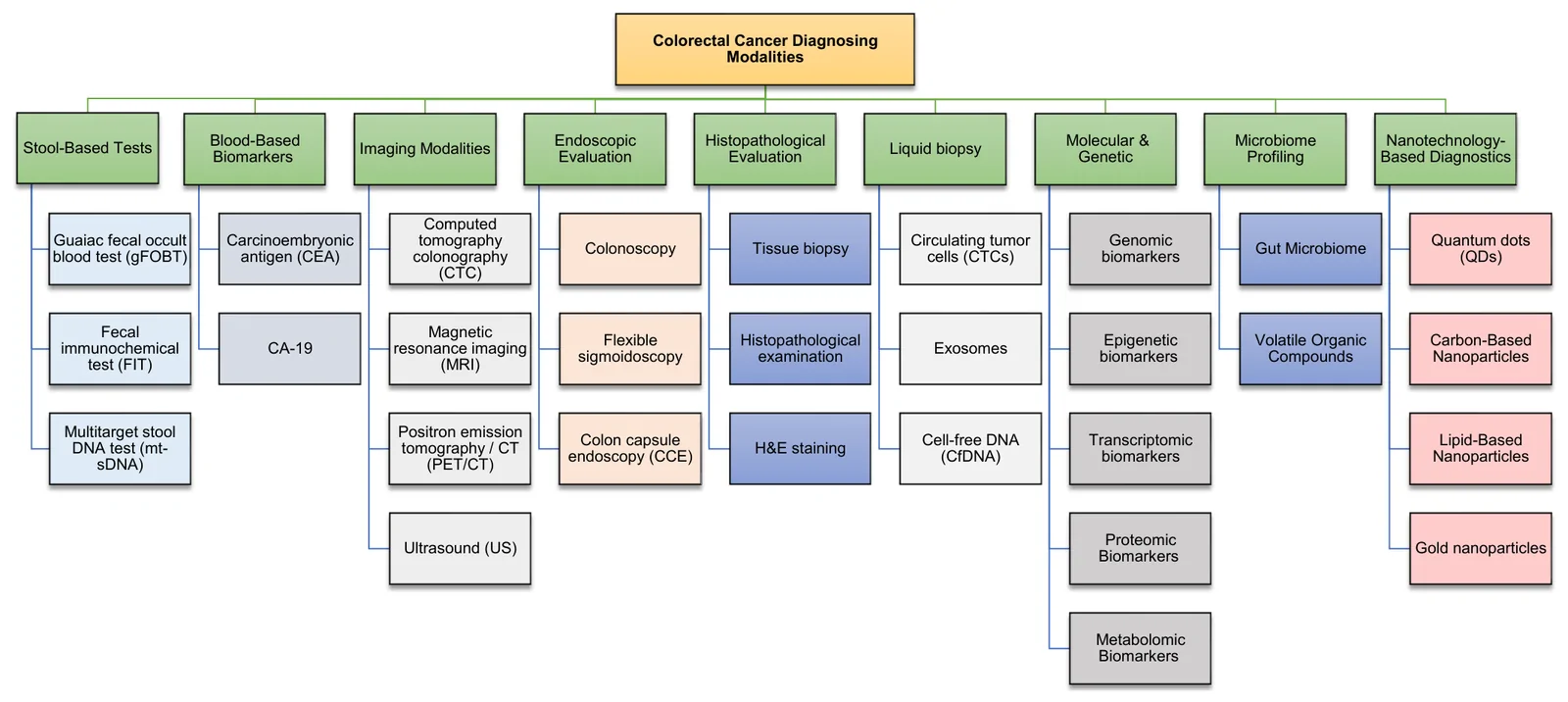
Taxonomy of colorectal cancer diagnosing modalities. The principal categories encompass stool-based screening tests, blood-based biomarkers, endoscopic visualization techniques, cross-sectional and functional imaging, histopathological tissue analysis, molecular and liquid biopsy assays, and emerging nanotechnology-based diagnostic platforms.

Diagnosis of CRC includes non-invasive screening, blood-based biomarkers, imaging, direct visualization, tissue analysis, and molecular profiling (refer to Figure 2). Screening identifies those who are at risk; imaging and endoscopy find the disease and determine the stage it has reached. Histopathology confirms malignancy, and molecular profiling directs treatment. We present a detailed taxonomy that classifies the main colorectal cancer diagnostic modalities into nine complementary categories (see Figure 3): stool-based tests, blood-based biomarkers, imaging modalities, endoscopic evaluation, histopathological evaluation, liquid biopsy, molecular and genetic, microbiome, and nanotechnology-based diagnostics.

### 3.1. Clinical Evaluation and Risk Stratification

Before any imaging, endoscopic, or laboratory test is deployed, clinical evaluation establishes the pre-test probability of colorectal neoplasia. The clinical assessment branch precedes all the other diagnostic modalities in Figure 2 and determines which downstream tests are indicated. Risk stratification begins with a structured history. Age is the strongest population-level risk factor, and most screening programs initiate at 45 to 50 years for average-risk individuals [3,29]. A family history of colorectal cancer or advanced adenoma in a first-degree relative before age 60 increases risk and warrants earlier and more frequent colonoscopic surveillance [2]. Hereditary syndromes, including Lynch syndrome and familial adenomatous polyposis, confer markedly elevated lifetime risk and require specialized surveillance protocols beginning in adolescence or early adulthood.

Inflammatory bowel disease, particularly ulcerative colitis and Crohn colitis of long duration, increases colorectal cancer risk and necessitates surveillance chromoendoscopy or high-definition white-light imaging (WLI) colonoscopy at regular intervals [30]. Lifestyle and metabolic factors, including obesity, type 2 diabetes, tobacco use, and heavy alcohol consumption, further modify risk and inform the choice of screening modality and interval. Symptomatic presentations, including rectal bleeding, iron-deficiency anemia, unexplained weight loss, and change in bowel habits, elevate the pre-test probability sufficiently to bypass screening tests and proceed directly to diagnostic colonoscopy with or without cross-sectional imaging [31]. Clinical risk scores and predictive models that integrate demographic, laboratory, and symptom data are increasingly used to triage patients toward the most appropriate diagnostic pathway. Artificial intelligence models that incorporate electronic health record data, complete blood count indices, and symptom clusters have shown promise in identifying patients who warrant urgent colonoscopic evaluation [32]. Table 4 summarizes the principal risk categories and their recommended diagnostic pathways.

### 3.2. Fecal Occult Blood Test

Stool-based tests offer a simple and inexpensive screening method by detecting microscopic blood traces that tumors leak into the intestinal lumen [1]. This subsection corresponds to the stool-based tests branch of Figure 3, which includes the guaiac fecal occult blood test (gFOBT), the fecal immunochemical test (FIT), and the multitarget stool DNA test (mt-sDNA). The two primary types are guaiac-based tests and fecal immunochemical tests. While screening with these methods reduces colorectal cancer mortality, with a meta-analysis of six Hemoccult trials reporting a 16 percent reduction [33], the overall sensitivity for advanced polyps remains low at approximately 42 percent [33]. Sample collection challenges further limit their clinical utility [34].

The guaiac fecal occult blood test detects hidden blood via the pseudoperoxidase activity of heme. Patients smear a stool sample onto guaiac paper, where hemoglobin triggers an oxidative reaction that turns the paper blue [2]. Although inexpensive, widely available, and simple to administer, it only reduces colorectal cancer mortality by 15 to 33 percent [35]. Sensitivity is approximately 11 percent for advanced adenomas and 12 percent for carcinomas, respectively [35] (these values represent per-study point estimates as summarized in the cited European screening review and reflect the gFOBT test arm reported in the meta-analyzed trials). The test also lacks specificity. Upper gastrointestinal bleeding, antiplatelet medications, and certain foods cause false positives, requiring patients to follow strict dietary restrictions [1].

Fecal immunochemical testing relies on the detection of the globin component of hemoglobin using monoclonal or polyclonal antibodies [36]. The Lee et al. meta- analysis [37] reported a pooled sensitivity of 79 percent and specificity of 94 percent for colorectal cancer detection with one-sample quantitative FIT and substantial heterogeneity across the included cohorts; the Mousavinezhad et al. meta-analysis [38] confirmed that FIT outperforms gFOBT on participation, positivity, and false-negative rate without any dietary restrictions. Sensitivity for advanced adenomas is substantially lower than for carcinoma (approximately 25 percent in the same meta-analyses), which is why the test is best regarded as a carcinoma-detection rather than advanced-neoplasia-detection assay. It yields fewer false results, which results in higher screening participation rates and a 22 percent reduction in colorectal cancer incidence. Many tests use colloidal gold immunoassays, whose antibodies conjugated to nanoparticles bind specifically to hemoglobin for rapid detection with more than 80 percent sensitivity and specificity [39]. The main limitation remains higher cost.

Multitarget stool DNA testing offers another non-invasive option by detecting human hemoglobin alongside tumor-associated DNA biomarkers. These include KRAS mutations, aberrant methylation of the BMP3 and NDRG4 promoter regions, and beta-actin [40]. A proprietary algorithm combines the immunochemical assay and DNA results into a single outcome with a recommended screening interval of three years for average-risk individuals [40]. While this test has a higher false-positive rate than fecal immunochemical testing and both methods show low sensitivity for advanced adenomas [2], the DNA test excels at detecting actual malignancies. One study detected 60 of 65 colorectal cancers, yielding a sensitivity of 92.3 percent for the multitarget stool DNA test in the cited screening cohort [41]. Table 5 summarizes the primary stool-based screening tests. Sensitivity values, recommended screening intervals, and cost tiers allow clinicians to select the most appropriate test for their patient population.

### 3.3. Imaging Modalities

Imaging studies provide information about the structure and function of the colon and surrounding tissues that help clinicians to stage the disease, detect metastases and assess treatment responses (Figure 3, imaging modalities branch). The chosen modality depends on the clinical question being asked and the patient factors. Diagnosis and staging are routinely done with computed tomography (CT) and magnetic resonance imaging (MRI), while positron emission tomography combined with CT (PET/CT) and ultrasound (US) are used for follow-up. In the imaging modalities branch of Figure 3 are CT colonography, MRI, PET/CT and US. CT gives a wide evaluation of the abdomen and distant metastases. MRI provides detailed images of the pelvis and soft tissue. PET/CT detects metabolically active lesions, and US is a non-invasive method of assessment.

In CT colonography, the digital image data are processed to enhance the density difference between tissue and gas. This produces two- and three-dimensional intraluminal images. This reconstruction allows the clinician to identify polyps without the insertion of a colonoscope and is an alternative when traditional colonoscopy is contraindicated, incomplete or poorly tolerated. The lack of required sedation supports its use as a screening modality, which is recommended for average-risk adults every 5 years by the American College of Gastroenterology [29,42,43]. The original large-scale validation of CT colonography for screening was reported by Pickhardt et al. [44], who demonstrated per-patient sensitivities of 88–94% for adenomas ≥ 6 mm and 96% sensitivity for adenomas ≥ 10 mm; the long-term programmatic experience is reported in the more recent Pickhardt et al. [43] review. The procedure requires colonic distension by air insufflation and cathartic bowel preparation [45]. Oral contrast agents can be used to aid fecal tagging. It exposes patients to ionizing radiation, has lower sensitivity for flat neoplasia [46] and is limited by infrastructure and insurance coverage issues, limiting its use for routine screening.

Magnetic resonance imaging (MRI) creates high-resolution images using strong magnetic fields and radio-frequency pulses without the use of ionizing radiation [47]. A scanner of at least 1.5 Tesla field strength is necessary for rectal cancer protocols, and an external phased-array coil is recommended, although bowel preparation and spasmolytic agents are generally not required [48]. MRI is the method of choice for local staging of rectal cancer, determining the depth of tumor invasion into the rectal wall and adjacent structures and differentiating between sigmoid and rectosigmoid tumors and upper rectal tumors [49]. Radiologists assess tumor location, size, longitudinal extent, T-stage, and regional lymph node involvement. The scan also shows involvement of mesorectal fascia, the circumferential resection margin and the distance from the anorectal junction [50]. Advanced sequences provide functional data, for example diffusion-weighted imaging that assesses the diffusion of water molecules to better detect tumor and dynamic contrast-enhanced sequences that evaluate tumor vascularity and tissue perfusion to predict response to treatment [51]. It is limited by high costs, long examination times and incompatibility with certain metallic implants. The modality also has difficulty differentiating T2 tumors from early T3 tumors and may overstage due to desmoplastic fibrosis. Moreover, whole-body protocols require specialized equipment and trained radiologists, limiting their availability.

PET/CT is combined functional and anatomic imaging. The PET part uses gamma radiation from intravenously administered radiotracers, and the CT part provides detailed anatomical localization [52]. The standard radiotracer 18F-fluorodeoxyglucose measures glucose uptake to detect metabolically active tumors [53]. This hybrid scan is primarily used by clinicians to detect recurrent disease, determine occult metastases, restage patients and assess responses to treatment. The test can detect recurrence with approximately 90 percent sensitivity and greater than 70 percent specificity, as summarized in the cited review by Lund et al. [54], and it helps to identify synchronous lesions that are proximal to an obstruction when a full colonoscopy cannot be performed. However, the modality has limited utility in lymph node staging. The use of FDG-PET in primary tumor staging is also hampered by poor spatial resolution as the maximum standardized uptake value reflects metabolic activity and correlates better with tumor size than the exact T-stage [55,56]. The procedure has an effective radiation dose of about 6 to 7 millisieverts and takes about three hours. Hyperglycemia and diabetes mellitus lead to reduced diagnostic accuracy by decreasing cellular radiotracer uptake [52,57,58,59]. False negatives may happen in the case of small tumors and mucinous adenocarcinomas, whereas false positives may be due to physiological uptake and inflammation [59]. The high cost of scanners also limits availability in low- and middle-income countries.

Ultrasound uses high-frequency sound waves to produce real-time images of internal structures with a hand-held transducer directly applied on the skin [54]. The modality is widely available, inexpensive and free of ionizing radiation, which makes it safe for repeated examinations in all patient populations [60]. Transabdominal ultrasound (TAUS) has a limited role in the diagnosis of primary colorectal tumors compared to endoscopy, but rectal ultrasound has a larger role in local staging. It provides an assessment of the tumor depth of invasion with a sensitivity of 64–95% and an analysis of the perirectal tissues. Rectal ultrasound has been shown to be superior to MRI in differentiating T1 from T2 lesions in early rectal cancer with a pooled specificity of 86 percent compared with 69 percent for MRI in the meta-analysis by Bipat et al. [61]. The technique has limitations in evaluating regional lymph nodes and distant metastases, and accuracy is reduced following neoadjuvant chemoradiotherapy due to difficulty in differentiating treatment-induced tissue changes from a residual tumor, leading to overstaging. Novel techniques require more clinical validation before routine use. Contrast-enhanced endoscopic ultrasound (CE-EUS) with intravenous microbubble agents of sulfur hexafluoride has been used to assess tumor angiogenesis and a real-time vascular assessment, thus improving lesion characterization [62]. Multiplanar imaging and tumor volume measurement are feasible with three-dimensional rectal ultrasound, and elastography is used to measure tissue stiffness to discriminate between benign and malignant lesions [63]. Table 6 summarizes the main imaging modalities for diagnosis and staging of CRC. Clinicians can choose the modality to fit the clinical question based on ratings for radiation dose, sedation needs, soft tissue contrast, and availability.

Cross-Modality Comparative Synthesis: The four imaging modalities differ fundamentally in the type of diagnostic information they provide and the clinical scenarios for which they are best suited. CT colonography offers high spatial resolution for luminal polyp detection and is the only cross-sectional alternative to optical colonoscopy for primary screening, but it provides no functional tissue characterization and exposes patients to ionizing radiation. MRI delivers superior soft-tissue contrast without radiation, making it the reference standard for local rectal cancer staging and mesorectal fascia assessment; however, its long acquisition times and susceptibility to motion artifacts limit its utility for whole-abdomen metastatic surveys. PET/CT uniquely combines metabolic and anatomic information, enabling detection of occult metastases and treatment response assessment that are invisible on purely anatomic modalities, but its poor spatial resolution renders it unsuitable for primary tumor T-staging or small polyp detection. Ultrasound provides real-time radiation-free assessment at the point of care and excels at differentiating early T-stage rectal lesions, but its operator dependence and limited field of view restrict its role to local staging adjuncts rather than comprehensive disease evaluation. In practice, these modalities are complementary rather than interchangeable: CT colonography serves as a screening alternative, MRI anchors local staging, PET/CT surveys distant disease, and ultrasound supplements early rectal tumor assessment.

### 3.4. Direct Visualization

Endoscopic procedures provide physicians with the ability to inspect the colonic mucosa directly, permitting tissue sampling and immediate therapeutic interventions. This subsection corresponds to the endoscopic evaluation branch of Figure 3 and includes colonoscopy, flexible sigmoidoscopy and colon capsule endoscopy (CCE). The choice of a technique depends on the extent of the examination and the clinical condition of the patient. The reference standard for colorectal cancer detection is a colonoscopy. The procedure uses a flexible colonoscope equipped with a high-definition camera to visualize the whole colon, allowing tissue biopsy and therapeutic interventions, such as polypectomy [31]. Patients are required to undergo a bowel preparation and generally receive intravenous sedation. The 15 to 45 min exam has high diagnostic accuracy. Screening colonoscopy reduces colorectal cancer mortality by 68 to 88 percent across the studies reviewed by Kahi et al. [64]. One study reported a 29 percent reduction in overall mortality and a 47 percent reduction in mortality due to distal cancer but no significant reduction in mortality due to proximal cancer [65]. The ability to diagnose and treat lesions in a single visit makes this the preferred modality. Removal of precancerous lesions immediately prevents disease progression and supports a 10-year screening interval for average-risk individuals after a normal examination [2]. Physicians are also able to distinguish between adenomatous and hyperplastic polyps during the procedure [66]. Direct colonoscopy shows the highest sensitivity and specificity of the available screening tests. However, it is invasive and carries risks such as bowel perforation and significant bleeding, with major complications occurring in one to two cases per 1000 procedures [67,68]. The test is expensive, resource intensive and highly dependent on the skill of the endoscopist.

Flexible sigmoidoscopy is a procedure to visualize the rectum, sigmoid colon and descending colon using a short endoscope [69]. Tissue sampling and polypectomy are only allowed for distal left-sided lesions as the procedure does not evaluate the entire colon. The exam is faster and less invasive than a full colonoscopy and typically requires no conscious sedation. The method has a lower risk of bleeding and perforation and is cheaper and needs only enema preparation [70]. Once abnormalities are found, a follow-up full colonoscopy is needed to look at the rest of the bowel. The main limitation is the inability to reach the proximal colon, missing right-sided lesions and providing no mortality benefit for proximal cancers [70]. The current guidelines recommend this test every 5 years alone or every 10 years with annual fecal immunochemical testing [29]. Long-term studies have shown that it reduces the incidence of cancer by 20 percent and mortality by 27 percent across the trials reviewed by Ko et al. [70]. But the test is still not widely used in the United States as colonoscopy has a higher diagnostic yield and patients often prefer procedures to be performed under sedation [29].

Colon capsule endoscopy offers a minimally invasive option for screening. The patient swallows a disposable capsule containing two tiny cameras, light-emitting diodes, a battery and a wireless transmitter. Peristalsis moves the capsule through the gastrointestinal tract, and it captures high-resolution color images with an approximate 344-degree field of view [3]. This wireless transmission is received by an external recorder for review in the clinic. Tissue biopsies and therapeutic procedures are not feasible with the capsule [3]. While the first-generation device demonstrated moderate sensitivity for polyps greater than six millimeters [71], the second-generation device improved diagnostic accuracy, achieving 76.7 percent sensitivity and 90.7 percent specificity for advanced neoplasia measuring 10 mm or larger in the cited cohort [72]. Clinicians use this test when patients have an incomplete conventional colonoscopy or cannot undergo the standard procedure for other reasons. Regulatory agencies have not approved it for routine screening in average-risk individuals [72], and it is listed as an alternative option only by the American College of Gastroenterology. The procedure does not require sedation, bowel insufflation, or ionizing radiation [3]. However, the image quality is highly dependent on the preparation of the bowel, and interpretation requires special training [73]. In about one third of cases, the examination is incomplete because of delayed colonic transit or battery expiration and needs a follow-up conventional colonoscopy [3,73]. The test is expensive and contraindicated in patients with suspected bowel obstruction, strictures, swallowing disorders, or GI fistula [74]. The endoscopic methods used for direct visualization and tissue sampling are summarized in Table 7. The structured comparisons for procedural selection include biopsy capability, need for sedation, bowel preparation intensity, and recommended screening intervals. In Table 8, we present a comprehensive comparison of all the endoscopic and imaging modalities in terms of cost, procedure duration, invasiveness, radiation exposure, clinical indications, diagnostic agreement and error rates to aid in modality selection for a given patient context.

### 3.5. Histopathology

Tissue analysis is the definitive diagnosis of malignancy. Pathologists look at the cellular architecture and determine the type of tumor, the grade and the stage. This dictates all treatment decisions going forward. The branch of histopathological evaluation shown in Figure 3 includes tissue biopsy, histopathological examination and hematoxylin and eosin (H&E) staining. Histopathological examination is still the reference standard for definitive diagnosis [4]. Pathologists identify the structural and cellular abnormalities on biopsies or resected specimens from endoscopic or surgical procedures under the microscope [75].

Surgical specimens are processed in laboratories using standard procedures for accurate evaluation. The pathologist first examines the external surface for serosal involvement, circumferential margin status, tumor perforation and distant implants. Rectal resections also require an assessment of the integrity of the mesorectum [76]. The specimen is oriented, measured and inked at the circumferential margin. The pathologist opens the bowel along the antimesenteric border, avoiding the tumor, and notes the tumor location, size, and distance from the surgical margins [77]. The tissue is pinned out on a board and fixed in 10 percent neutral buffered formalin overnight for preservation of the morphology. The sample is dehydrated and cleared prior to embedding in paraffin. The paraffin blocks are then sectioned into approximately four-micron-thick sections using a microtome, which are mounted on glass slides by technicians [77].

Hematoxylin and eosin staining allows to see tissue architecture and cellular morphology. Microscopic examination confirms malignancy and assesses histological subtype, tumor grade, lymphovascular invasion, perineural invasion, and margin status. These results differentiate benign from malignant lesions and provide material for molecular analyses. Tissue acquisition is too invasive to be useful for primary screening. The accuracy of diagnosis depends on the adequacy of sampling, the expertise of the pathologist, and the specialized laboratory facilities [78]. Table 9 provides details of the standard workflow and key evaluation parameters in histopathological examination. The AI-readiness and automation columns identify the stages that are most amenable to computational augmentation.

### 3.6. Molecular Diagnostics

Molecular diagnostics analyze genetic and protein alterations associated with cancer to detect circulating biomarkers and characterize tumor biology (molecular and liquid biopsy branch, Figure 3). The results help clinicians to monitor disease progression and choose targeted treatments. Biomarkers are useful for the detection, diagnosis, prognosis and monitoring of colorectal cancer. Blood-based protein biomarkers include carcinoembryonic antigen (CEA) and carbohydrate antigen 19-9 (CA19-9) and are one of the types of blood-based biomarkers shown in Figure 3. These markers are routinely used by clinicians to monitor diseases. The molecular and liquid biopsy branch in Figure 3 comprises circulating tumor cells (CTCs), exosomes and genomic, epigenetic, transcriptomic, proteomic and metabolomic biomarkers. Genomic biomarkers can provide insight into tumor characteristics and predict treatment responses. Microfluidic technologies allow rapid analysis of these biomarkers in small blood volumes.

CEA is a glycoprotein that plays a role in cell adhesion and is the most frequently used protein biomarker in colorectal cancer. Marker is found in embryonic tissue and colorectal malignancies. Marker has a half life of two weeks. Its usefulness in primary diagnosis is limited because benign inflammatory conditions also cause an increase in these levels. The test remains useful in determining prognosis, response to treatment and recurrence [79]. CA19-9 is a serum protein of limited diagnostic value. The test has low sensitivity, especially in early-stage disease, and high specificity, making it unsuitable for population screening [42]. Microfluidics is the manipulation of small volumes of fluids in small channels and forms the basis of lab-on-a-chip devices used for rapid disease monitoring. The method is non-invasive, highly sensitive and reduces reagent consumption [80]. It analyzes circulating tumor cells, cell-free DNA, exosomes and microRNA. However, the lack of standardization and large-scale clinical validation at present has hampered routine clinical application.

Circulating tumor cells (CTCs) are released from primary or metastatic lesions into the circulation. Microfluidic platforms were employed to isolate these cells using immunoaffinity capture based on surface-immobilized antibodies [81]. These platforms are sensitive and specific and can be used to monitor disease progression in real time [82]. However, the capture efficiency is limited by the low abundance and biological heterogeneity of the cells. Cell-free DNA (cfDNA) is a circulating biomarker released into the bloodstream. Tumor-derived cfDNA is known as ctDNA. In CRC, ctDNA enables non-invasive molecular characterization in early detection and recurrence monitoring [83]. Exosomes derived from tumors are emerging biomarkers. Microfluidic platforms use selective antibodies for fast and high-throughput exosome detection [84]. Improving capture efficiency and reducing non-specific binding are still active areas of research [81]. Technological advances have accelerated the discovery of genomic, epigenetic, transcriptomic, proteomic and metabolomic biomarkers, providing new opportunities for molecular characterization [85].

Genomic biomarkers are the particular gene mutations that cause tumor formation. The APC tumor suppressor is responsible for controlling cell division via the Wnt signaling pathway, and mutations occur in 85 percent of colorectal cancers, one of the earliest genomic changes [86,87]. The TP53 tumor suppressor regulates cell-cycle progression and apoptosis. Mutations are found in 22 to 70 percent of cases and correlate with tumor progression and chemotherapy resistance [88,89]. KRAS is a proto-oncogene that controls cellular proliferation, and some mutations are highly correlated with tumor invasion and lymphatic metastasis [90,91]. The BRAF oncogene is involved in the regulation of cell differentiation, and 10% of colorectal cancers contain mutations that are important prognostic biomarkers [92]. The PIK3CA mutation is present in 10-15% of cases and is a potential target for therapy [85,93]. Finally, SMAD4 acts as a mediator of the transforming growth factor-beta pathway, and mutations are associated with metastasis and poor prognosis [94].

Epigenetic biomarkers are the indicators of aberrant gene regulation in tumorigenesis. DNA methylation is the most widely studied alteration. Potential for early detection exists in methylated genes like NDRG4, BMP3, SEPT9 and SDC2. SEPT9 is an established blood-based biomarker, and NDRG4 and BMP3 are common components of stool DNA tests. Large-scale validation studies are required for a widespread clinical implementation [95]. Transcriptomic biomarkers are representative of RNA expression alterations. The majority of the human genome is transcribed into non-coding RNAs [96]. Technologies such as microarray and sequencing allow for comprehensive profiling of these molecules [97]. MicroRNAs are small non-coding RNAs that regulate mRNA degradation and translation. Dysregulated expression of microRNAs is consistently observed in colorectal cancer, and they are useful minimally invasive biomarkers [98]. Long non-coding RNAs are transcripts composed of more than 200 nucleotides. Their abnormal expression promotes colorectal cancer progression and offers targets for diagnosis and therapy [99]. Circular RNAs are robust single-stranded molecules with covalently linked ends and have shown promise for early diagnosis with altered expression, although further clinical validation is pending [100].

Proteomic biomarkers are large-scale studies of protein profiles. Differentially expressed proteins are identified using mass spectrometry, chromatography, and protein microarrays [101]. Colorectal cancer is characterized by altered expression of a variety of proteins that are implicated in tumor progression and metastasis. Large-consortium projects continue to expand the discovery of therapeutic targets. However, proteomics detects low-abundance proteins but needs more validation before routine clinical use. Metabolomic biomarkers are based on the global analysis of small-molecule metabolites. Metabolic reprogramming is a hallmark of cancer, and this profiling approach is highly relevant [102]. Liquid chromatography, gas chromatography and nuclear magnetic resonance spectroscopy are analytical platforms. Certain metabolomic signatures can separate patients from healthy subjects and predict overall survival [103]. One study reported a diagnostic accuracy of 97.2 percent using blood metabolomic biomarkers in the cited single-cohort analysis [104], although further validation studies are needed before clinical implementation. Table 10 presents the molecular and biomarker assays utilized in the management of colorectal cancer. Validation status and clinical readiness ratings help clinicians to differentiate between established tests and those still in the research phase.

### 3.7. Gut Microbiome and Volatile Organic Compounds

The gut microbiome and volatile organic compound profiles are gaining recognition as non-invasive diagnostic inputs for colorectal cancer detection. These modalities extend the molecular and liquid biopsy branch of Figure 3 by capturing metabolic and microbial signatures associated with colorectal carcinogenesis. Certain bacterial taxa are enriched in the stool and colorectal tumor tissue compared with healthy controls, including Fusobacterium nucleatum, Bacteroides fragilis, and some strains of Escherichia coli. Machine learning classifiers trained on 16S ribosomal RNA sequencing or shotgun metagenomic data have been shown to be able to differentiate colorectal cancer patients from healthy individuals with moderate to high accuracy [32]. These microbial signatures are complementary to the existing stool-based tests and may enhance sensitivity for early-stage lesions that shed insufficient hemoglobin or DNA to be detected by FIT or multitarget stool DNA assays.

Volatile organic compounds are small-molecule metabolites excreted in breath, urine or stool as by-products of altered tumor metabolism and microbial fermentation. These composite profiles are recorded by electronic nose devices and gas chromatography–mass spectrometry platforms. Artificial intelligence models trained on signatures of volatile organic compounds have shown promise as non-invasive screening tools, especially in populations with low stool collection or colonoscopy acceptance [32]. Microbiome- and volatile organic compound-based approaches are still at the research and early validation stage. There is a need to standardize sample collection, sequencing protocols and compound identification pipelines for clinical deployment. Sensitivity, specificity and acceptable false-positive rates for population screening will need to be established by prospective validation against the reference standard of colonoscopy in large samples.

### 3.8. Nanotechnology

Nanotechnology with the use of molecular-scale materials increases diagnostic sensitivity. These platforms amplify biological signals and allow for multiplexed testing. Research continues to translate these tools from the laboratory to the clinic. In the nanotechnology-based diagnostics branch, as shown in Figure 3, we have quantum dots (QDs), carbon-based nanoparticles, lipid-based nanoparticles, and gold nanoparticles. Nanotechnology enhances detection of low-abundance biomarkers that are undetectable by traditional means. Nanomaterials have a high surface-area-to-volume ratio and excellent signal amplification properties [106]. These features help to facilitate early detection and reduce false positives. The platforms [107] offer multiplex analysis, meaning the ability to detect multiple biomarkers in blood, stool and tissue samples at the same time. Such versatility is useful for early cancer detection, liquid biopsy, and molecular imaging. Most nano-enabled platforms are either in the research or early validation phase. Reproducible fabrication, long-term biocompatibility concerns and need for specialized equipment limit widespread clinical adoption [107].

Fluorescent semiconductor nanoparticles, known as quantum dots (QDs), exhibit high brightness and photostability. Researchers attach specific targeting ligands to selectively detect cancer-associated biomarkers. These particles are highly sensitive for circulating tumor cell detection and molecular imaging, but toxicity issues and limited clinical validation still pose major challenges [108]. Carbon-based nanoparticles comprise carbon nanotubes, graphene, and carbon dots. These materials exhibit high biocompatibility, large surface area and suitable electrical conductivity for biosensor applications [109]. Surgeons use them for sentinel lymph node mapping to increase staging accuracy. Carbon nanoparticle-guided biopsy was reported to be 91.67 percent sensitive and 100 percent specific in detecting lymph node metastasis in the cited single-center study by Yan et al. [110].

Lipid-based nanoparticles are composed primarily of phospholipids or cholesterol. They possess excellent biocompatibility, stability and strong targeting ability. Formulators conjugate particular ligands for biomarker recognition and encapsulate imaging agents to improve lesion visualization [111]. Their clinical translation is currently limited by formulation challenges and regulatory hurdles despite promising preclinical results. Gold nanoparticles are noble-metal nanostructures that possess high biocompatibility, tunable surface chemistry and strong optical contrast. They can be functionalized with antibodies, peptides or probes of nucleic acids to target colorectal tumor markers and are being investigated for molecular imaging, biosensing and photothermal applications. Standardized synthesis, long-term biodistribution data and regulatory validation are still required prior to routine clinical use. Table 11 lists the emerging nanotechnology platforms applied to colorectal diagnostics. Biocompatibility ratings and maturity levels show which platforms are the closest to clinical translation.

Relevance to artificial intelligence: The nanotechnology platforms described above intersect with artificial intelligence at three points. First, nanoparticle-enhanced biosensors generate multiplexed signal arrays that require machine learning classifiers for pattern recognition and biomarker deconvolution; conventional threshold-based readouts are insufficient when six or more analytes are measured simultaneously [107]. Second, quantum-dot and gold-nanoparticle molecular imaging produces high-dimensional fluorescence or photoacoustic data whose interpretation benefits from deep learning segmentation and classification pipelines that are analogous to those described in Section 4. Third, microfluidic lab-on-a-chip devices that isolate circulating tumor cells or exosomes (Section 3) generate image and signal streams that are amenable to convolutional or recurrent neural network processing [80]. However, no included study in this review reported a prospective AI-nanotechnology diagnostic trial for colorectal cancer. The evidence remains preclinical or early-phase. Claims of diagnostic sensitivity or specificity for nano-enabled platforms should therefore be interpreted as exploratory until validated in clinical cohorts with appropriate reference standards.

## 4. Artificial Intelligence Frameworks for Colorectal Diagnosis

**Figure 4:**
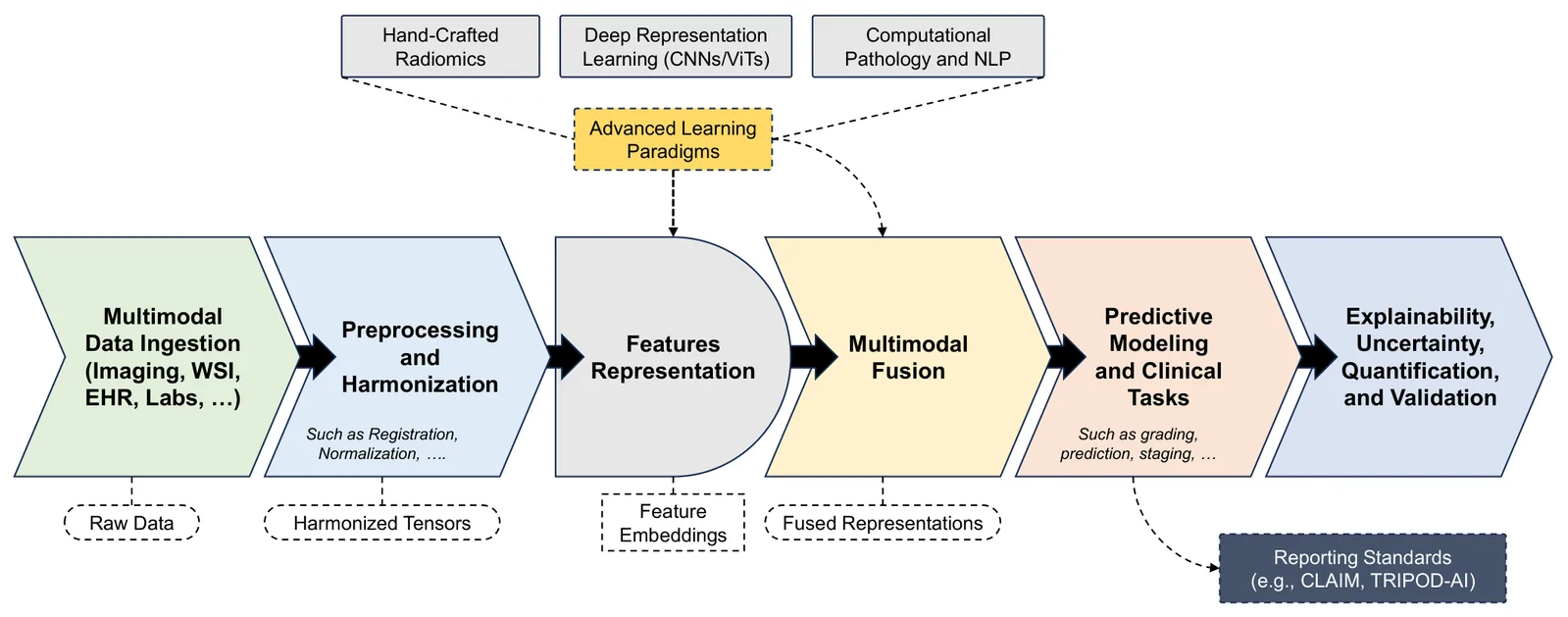
Staged artificial intelligence framework for colorectal diagnosis. Chevron stages map the methodological toolkit (radiomics, deep representation learning, computational pathology, and natural language processing) onto the diagnostic pipeline. Dashed satellites indicate the advanced learning paradigms that condition representation and fusion and the reporting standards (e.g., CLAIM and TRIPOD-AI) that govern modeling and validation.

Clinical, endoscopic, radiological, and pathological data, when integrated into clinical decision-making, requires rigorous artificial intelligence frameworks [10,25,112,113,114]. The modern toolkit in colorectal care is built upon hand-crafted radiomics pathways, deep segmentation architectures, computational pathology frameworks, natural language processing strategies, and advanced learning paradigms. The conceptual organization of these components into a staged diagnostic framework, including the data objects transferred between stages and the governance modules that condition them, is summarized in Figure 4.

### 4.1. The Comprehensive AI Pipeline in Colorectal Care

**Figure 5:**
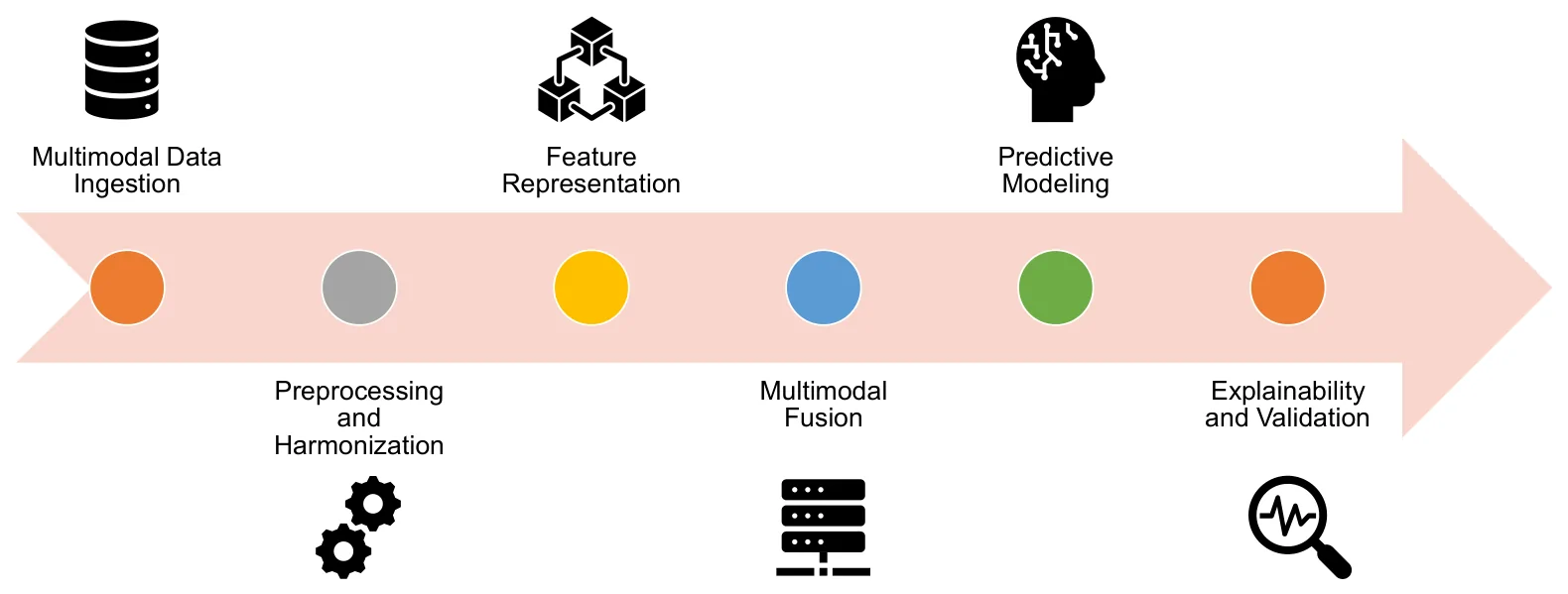
The comprehensive artificial intelligence pipeline for colorectal diagnosis. The pipeline comprises multimodal data ingestion, preprocessing and harmonization, feature representation, multimodal fusion, predictive modeling, and explainability and validation.

The development of artificial intelligence for colorectal diagnosis consists of a multi-stage pipeline to minimize domain shift and ensure clinical validity. First, ingest and harmonize data. We need to standardize formats such as DICOM for cross-sectional imaging, SVS for whole-slide images, and proprietary colonoscopy video streams. Next is preprocessing, consisting of intensity normalization, spatial resampling, histology stain normalization and artifact correction. The regions of interest are isolated, features are represented, forecasting is performed, and the final calibration is done on the validation set. This development and validation life cycle is formalized in Algorithm 1. It is important to emphasize that Algorithm 1 is not a bespoke software implementation or executable code; rather, it is a conceptual synthesis constructed for the purposes of this review to illustrate, compare, and contrast the methodological patterns observed across the 160 included references. The algorithm serves as a schematic checklist that maps the most frequently reported workflow stages onto a unified pipeline representation. The conceptual stages of this pipeline are shown in Figure 5: multimodal data ingestion, preprocessing and harmonization, feature representation, multimodal fusion, forecasting, and explainability and validation.

Evidence basis and validation status: The pipeline presented above is a conceptual synthesis of methodological patterns across the included studies, constructed specifically for this review to enable cross-study comparison and synthesis. This is not a bespoke software implementation, a prospectively validated framework, or executable code. The staging sequence (ingestion, preprocessing, feature representation, fusion, modeling, explainability, and validation) mirrors the most frequently reported order of the workflow in the reviewed literature but has not been empirically evaluated as a unified protocol. Assumptions: The pipeline assumes (i) sufficient labeled data is available for supervised training, (ii) a reference standard is available for validation, (iii) computational resources are available for the described preprocessing and model training, and (iv) domain shift between training and deployment sites can be minimized by the described harmonization steps. These assumptions may not hold in low-resource settings, rare disease contexts or prospective screening programs.
**Algorithm 1:** Conceptual synthesis of the colorectal-specific multimodal artificial intelligence diagnostic pipeline, constructed from methodological patterns across the reviewed literature for the purpose of review, comparison, and pedagogical illustration. The pipeline incorporates colonoscopy video frame extraction, whole-slide-image patch extraction, DICOM harmonization across CT and MRI, and modality-missing handling. This is not a bespoke implementation or executable code
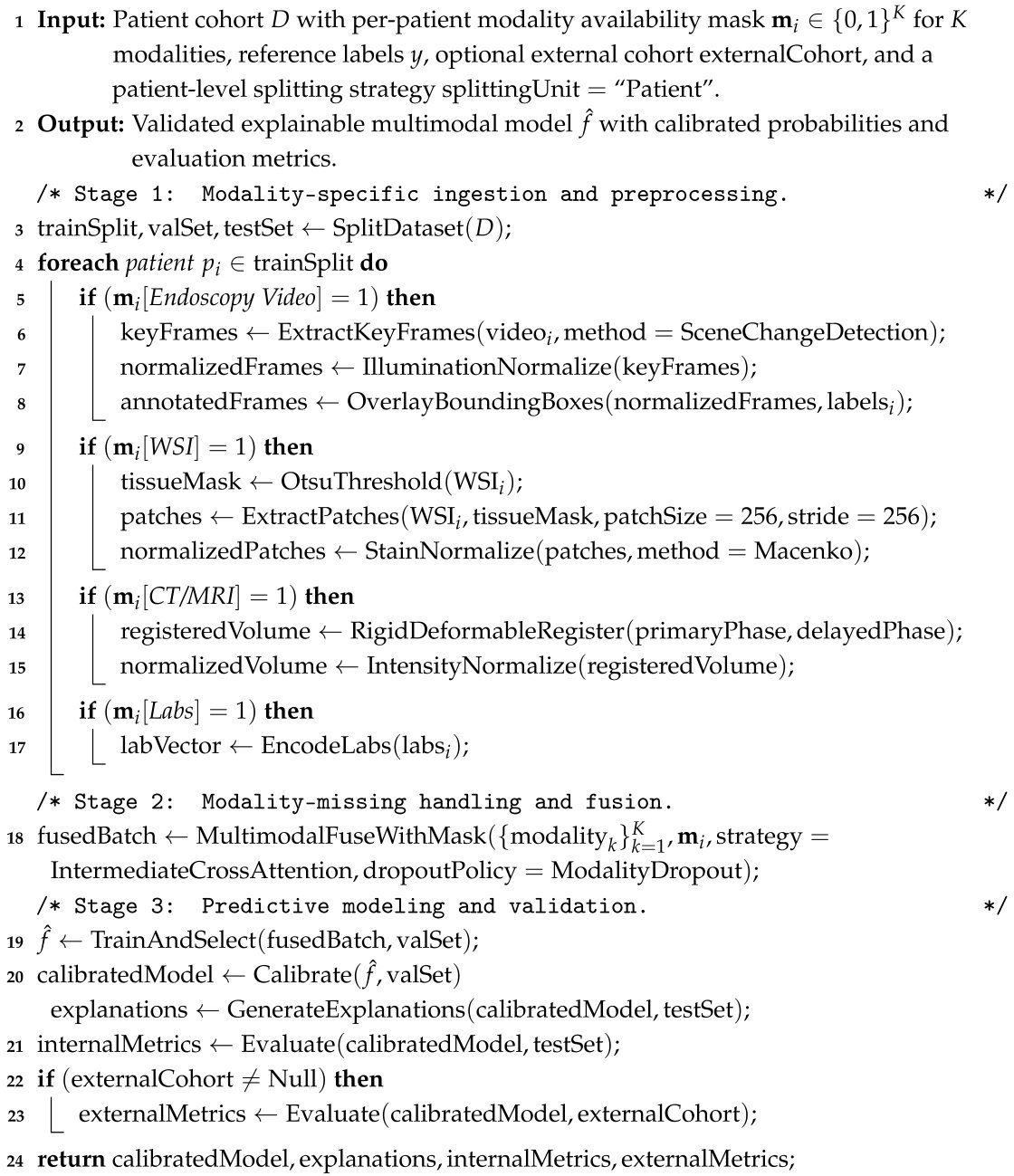


Limitations: The pipeline does not specify specific architectures, hyperparameters, or sample sizes. It does not consider regulatory submission requirements, real-time inference latency constraints, or post-deployment monitoring protocols other than the MLOps discussion in Section 7. Readers should view Algorithm 1 as a schematic checklist rather than executable code. Clinical Case Scenario (Hypothetical): The following is an illustrative scenario. It does not describe a validated or deployed clinical decision support system. All the patient details are fictitious. A 55-year-old patient with a positive fecal immunochemical test undergoes clinical evaluation. The artificial intelligence pipeline ingests the electronic health record, normalizes prior colonoscopy video frames, and harmonizes the new biopsy whole-slide image. The system flags a high-risk adenoma and outputs a calibrated probability on the validation set.

### 4.2. Hand-Crafted Features and Radiomics in Cross-Sectional Imaging

Hand-crafted feature extraction remains a fundamental framework for quantification of imaging biomarkers in CT colonography and MRI [13,47]. For cohorts with a small number of observations, it is preferable to decouple feature extraction from predictive models to improve interpretability. The radiomics workflow starts with accurate segmentation of the primary tumor, colon wall and lymph nodes of interest. Mathematical descriptors are extracted from the region of interest. These features are first-order statistics that describe the distributions of voxel intensities and shape descriptors that describe the sphericity or surface irregularity of the tumor. Higher-order texture matrices contain information on spatial spread and heterogeneity. The gray-level co-occurrence matrix and the gray-level run-length matrix were used to quantify the mucinous components and desmoplastic stromal reactions of colorectal malignancies. This subvisual textural information is helpful in distinguishing early T-stage rectal tumors and predicting microscopic extramural vascular invasion. Manual visual assessment of such tasks is prone to high inter-observer variability. Strict adherence to the guidelines of the Image Biomarker Standardization Initiative guarantees reproducibility across institutions. Feature selection frameworks like Least Absolute Shrinkage and Selection Operator extract the most discriminative subsets. Subsequently, traditional machine learning classifiers are trained on the reduced feature space for classification or survival regression.

Clinical Case Scenario (Hypothetical): A 62 year old woman is diagnosed to have cancer of the rectum. Preoperative magnetic resonance imaging reveals a tumor close to the mesorectal fascia. A radiomics model trained on similar data could extract texture features from the tumor periphery that are undetectable to the human eye. The model could predict extramural vascular invasion with high accuracy. This prediction may lead the surgical team to consider neoadjuvant chemoradiotherapy instead of immediate surgery.

### 4.3. Deep Representation Learning for Endoscopic and Pathological Imaging

Deep learning frameworks employ artificial neural networks to learn hierarchical feature representations from raw pixel or video data. Segmentation of anatomical structures from complex backgrounds is a prerequisite for many downstream tasks. Correct identification of polyps and rectal tumors is the basis for volumetric assessment and surgical planning. This also allows for localized feature extraction [28,115]. The U-Net model with symmetric encoder–decoder structure and skip connections remains the gold standard in biomedical image segmentation. More advanced variants include spatial attention gates that suppress irrelevant background regions and focus on focal lesions. Colonoscopy videos present a unique set of challenges [116,117]. These include motion blur, specular reflections and changing illumination. Poor bowel preparation complicates analysis. The separation of true polyps from transient artifacts, such as folds or bubbles, is achieved by entering temporal sequences to three-dimensional convolutional networks and recurrent neural networks. For 3D volumetric data from magnetic resonance imaging, architectures such as V-Net are used to model spatial dependency between slices for segmentation of rectal tumors and mesorectal fascia.

Once the region of interest is identified, deep representation learning frameworks connect the imaging data to clinical endpoints. Stacked convolutional filters extract low-level edges and high-level semantic patterns in convolutional neural networks. Vision transformers overcome the limitations of convolutional neural networks in modeling long-range spatial dependencies. These models are based on splitting the image into fixed-size patches and operating on them with multi-head self-attention mechanisms. This enables the model to incorporate global contextual information across the whole endoscopic frame or pathology slide. These architectures are currently used by computer-aided diagnosis systems for optical diagnosis in real time. This capability underpins endoscopists’ confident application of leave-in situ strategies for diminutive rectosigmoid polyps [16,118,119,120,121].

Clinical Case Scenario (Hypothetical): A 48-year-old man undergoes a screening colonoscopy that finds a 4 mm polyp in the sigmoid colon. A deep learning model analyzes the endoscopic video feed in real time. The vision transformer architecture analyzes the vascular pattern, confidently classifying the polyp as hyperplastic. The endoscopist leaves the polyp in place. The patient is spared the risks and costs of unnecessary polypectomy and pathology.

### 4.4. Computational Pathology and Weakly Supervised Frameworks

Histopathology provides ground truth at the cellular level but is computationally challenging to scale [122]. Whole-slide images can be larger than 100,000 × 100,000 pixels. This size makes it impossible to ingest it directly into standard convolutional neural networks. Computational pathology frameworks tackle this limitation with patch-based processing pipelines. It is too expensive to acquire pixel-level annotations for gigapixel whole-slide images at scale. Multiple Instance Learning is how weakly supervised frameworks tackle this problem. In this paradigm, a whole-slide image is considered as a bag containing a large number of smaller image patches. The diagnostic label is provided at the bag level only. The framework incorporates instance-level features into a bag-level prediction through attention-based pooling mechanisms. This process finds the most diagnostically relevant tissue regions without requiring extensive manual annotations [10]. This method is very helpful in colorectal cancer for quantification of the tumor budding and assessment of spatial distribution of tumor-infiltrating lymphocytes. It also aids in evaluating the entire circumferential resection margin. Doing these tasks manually is tedious and prone to sampling bias.

Clinical Case Scenario (Hypothetical): A pathology lab receives a whole-slide image of a colon cancer resection specimen. Counting the tumor buds manually over the whole slide would take hours. The gigapixel image is divided into patches and processed by a Multiple Instance Learning framework. The model recognizes the case as high-risk for lymph node metastasis with high density of tumor buds at the invasive front. This information helps the oncologist to recommend adjuvant chemotherapy.

### 4.5. Natural Language Processing and Multimodal Fusion

Models limited to a single modality are constrained by the intrinsic information boundaries of a specific data source. Multimodal fusion frameworks fuse heterogeneous data streams into holistic patient representations. Fusion strategies work at the early or late levels. They can also operate at intermediate or hybrid levels. Intermediate fusion employs a cross-attention mechanism in a transformer architecture. This lets the model learn dynamically how important each feature is across the modalities. For example, a model may link magnetic resonance imaging radiomics of a rectal tumor to circulating tumor DNA levels. It may also include patient comorbidities to predict the likelihood of achieving a pathological complete response after neoadjuvant chemoradiotherapy. Natural language processing frameworks simultaneously extract structured phenotypic variables from unstructured clinical notes. Large language models process clinical histories and notes from multidisciplinary team meetings. These models connect qualitative clinical notes to quantitative artificial intelligence models. Large language model recommendations are highly concordant with traditional multidisciplinary team decisions for the management of colorectal cancer according to recent work [123]. This fusion process corresponds to the multimodal fusion and prediction modeling stages in Figure 5.

Clinical Case Scenario (Hypothetical): A multidisciplinary tumor board reviews a 70-year-old patient with locally advanced colon cancer and multiple comorbidities. An artificial intelligence system fuses the radiological tumor volume with the genomic mutation profile. Technical Limitations and Prerequisites: In routine practice, achieving this workflow requires complete multimodal data availability, which is often hindered if (missing genomic assays or misaligned imaging timestamps occur). Furthermore, the model must be robust to domain shift across different sequencing platforms and MRI vendors, and extraction biases from unstructured clinical notes must be mitigated via rigorous natural language processing validation.

### 4.6. Advanced Learning Paradigms for Data Constraints

Data scarcity, class imbalance and strict privacy regulations limit the development of robust artificial intelligence frameworks in colorectal care. Advanced learning paradigms overcome these limitations in the methodological pipeline. Models are initialized with weights pre-trained on large natural-image datasets or large-scale medical repositories in transfer learning. This greatly reduces dependence on large annotated cohorts. Generative adversarial networks and diffusion models synthesize realistic pathological variants to balance training distributions. Federated learning and swarm learning facilitate training models across multiple institutions by iteratively aggregating model gradients rather than sharing raw patient data. This enhances privacy and regulatory compliance and mitigates single-center selection bias.

Clinical Case Scenario (Hypothetical): A rare subtype of colorectal neuroendocrine tumor lacks sufficient local cases to train a reliable diagnostic model. Researchers use transfer learning to initialize a convolutional network with weights from a large general oncology dataset. They then fine-tune the model on a small federated cohort of rare tumors from five international hospitals without sharing patient data. The resulting model achieves high diagnostic accuracy despite severe data scarcity.

### 4.7. Explainability, Uncertainty Quantification, and Reporting Standards

The internal decision logic of deep and multimodal artificial intelligence models remains opaque to direct inspection. Explainable artificial intelligence serves two complementary goals. It increases clinician trust and provides auditability for safety-critical decisions. Explainability methods can be intrinsic or post hoc. In tabular and multi-omic models, Shapley Additive Explanations quantify the contribution of individual clinical or molecular features. This is particularly useful for identifying microbiome biomarkers in colorectal cancer [124]. In imaging, gradient-weighted class activation mapping identifies the regions that most influence classification. These methods are the most useful when they are task-specific and evaluated with clinician-in-the-loop studies.

Uncertainty quantification is the essential complement to explainability. Monte Carlo dropout, deep ensembles and bootstrap resampling are employed to identify low-confidence or out-of-distribution cases that require human review. Calibration tests provide another layer of safety. Reliability diagrams and expected calibration error metrics quantify the degree of alignment between predicted probabilities and observed outcomes. A model that is claimed to have 90 percent confidence but is only correct 60 percent of the time will mislead clinicians who use probability thresholds to triage patients. Explanation stability and uncertainty estimates should be reported alongside accuracy, area under the receiver operating characteristic curve and Dice similarity coefficients for clinical deployment. Auditable evidence is produced by standardized reporting of these technical safeguards. TRIPOD-based principles for artificial intelligence should be applied in reporting of studies of prediction models. Randomized trials should use CONSORT-AI and the Checklist for Artificial Intelligence in Medical Imaging, which provide modality-specific reporting for imaging and radiomics. A summary of the key reporting and governance frameworks is presented in Table 12. The tick and cross symbols enable readers to see which framework fills the particular gap in validation in their study design.

Clinical Case Scenario: An artificial intelligence tool flags a routine surveillance colonoscopy video as containing a high-risk sessile serrated lesion. The system provides a gradient-weighted class activation mapping overlay. This overlay highlights the specific mucus cap and dilated crypt openings that drove the prediction. The uncertainty quantification module reports a low confidence score due to poor bowel preparation in that segment. The calibration curve for this model shows that predictions in the 40 to 60 percent probability range are poorly calibrated, which means the reported score is unreliable. The endoscopist trusts the visual explanation but orders a repeat colonoscopy to confirm the finding.

## 5. Task-Specific Artificial Intelligence Studies in Colorectal Diagnosis

The implementation of artificial intelligence frameworks in the clinical practice requires extensive validation on specific diagnostic and prognostic tasks. This section reviews task-specific studies, including computer-aided detection, optical diagnosis, estimation of invasion depth, inference of molecular phenotypes, prediction of metastasis, detection of surgical phases, and human–artificial intelligence interaction. Performance indicators, clinical utility and translational barriers are described based on the current evidence for each application domain. The definition, threshold and validation protocol of performance indicators reported across the included studies are heterogeneous. The following conventions are employed throughout this section to avoid misleading comparisons between studies.

1. Within-task comparison only: Metrics are compared only among studies addressing the same clinical task on the same modality. Cross-task comparisons (e.g., polyp detection ADR versus metastasis AUC) are not made.
2. Metric definitions: Sensitivity and specificity refer to per-lesion or per-patient performance as stated in the original study. AUC refers to the area under the receiver operating characteristic curve unless otherwise noted. ADR is the adenoma detection rate per colonoscopy. Dice and IoU refer to segmentation overlap.
3. Validation protocol annotation: Each table row notes whether performance was measured on internal test, external, or prospective data. Metrics from different validation levels are not directly compared.
4. No pooled estimation without meta-analysis: Where fewer than three studies report the same metric on comparable populations, no summary estimate is computed. Narrative synthesis is used instead.

### 5.1. Computer-Aided Detection in Endoscopy

Computer-aided detection systems analyze video streams of white-light or narrow-band imaging in real time to define suspected polyps. These systems (refer to Table 13) have been shown in multiple randomized controlled trials to improve adenoma detection rates by reducing perceptual blind spots and fatigue-related misses. Repici et al. [5] reported a significant improvement in adenoma detection with real-time computer-aided detection compared to standard high-definition white-light colonoscopy. These findings were corroborated by Wang et al. in a prospective randomized study with increased polyp and adenoma detection rates [6]. These benefits are further supported by tandem colonoscopy trials. The CADeT-CS trial demonstrated that deep learning computer-aided detection significantly reduces the adenoma miss rate compared with routine white-light colonoscopy [125,126]. Similarly, Wallace et al. reported a significant reduction in the miss rate of colorectal neoplasia using artificial intelligence [127]. These findings are summarized in the systematic reviews and meta-analyses by Hassan et al. and Nazarian et al. The latter meta-analysis included 48 studies out of 899 records and confirmed the diagnostic accuracy of computer-aided diagnosis in the detection and characterization of colorectal polyps [27,128]. The effectiveness of these systems is additionally confirmed by Mehta et al. [28].

Beyond controlled trial environments, real-world implementation studies validate these performance gains. Ishiyama et al. conducted a large-scale propensity score-matched study showing that clinical use of artificial intelligence-assisted neoplasia detection sustains high adenoma detection rates in routine practice [129]. The technology also narrows the performance gap between novice and expert practitioners. Biscaglia et al. found that real-time computer-aided detection eliminates differences in adenoma detection rates between trainees and experienced endoscopists [130]. Furthermore, artificial intelligence mitigates the well-documented decline in procedural quality that occurs later in the day. Lu et al. and Richter et al. independently demonstrated that computer-aided detection preserves high adenoma detection rates throughout the clinical workday, effectively counteracting endoscopist fatigue [131,132]. Nakashima et al. evaluated a novel endoscopic artificial intelligence system in a single-center randomized controlled trial, confirming sustained improvements in neoplasia detection [133]. Gong et al. introduced the ENDOANGEL system, a real-time computer-aided detection platform that significantly increased adenoma detection in a single-center randomized study conducted at Renmin Hospital of Wuhan University [7]. Young et al. summarized the role of these systems in lesion detection, noting that continuous algorithmic feedback stabilizes procedural quality metrics across varying clinical conditions [115].

**Table 13:** Summary of computer-aided detection studies in endoscopic colorectal evaluation.

Study (Year)	Architecture/Modality	Clinical Task	Best Performance	Validation Level	Principal Limitation
Repici et al. (2020) [5]	CNN/White-light video.	Real-time polyp detection.	ADR: 54.8% vs. 40.4% (RR 1.30, *p* < 0.001).	Prospective multicenter RCT.	Trial-specific population.
Wang et al. (2019) [6]	Deep learning/Video.	Polyp and adenoma detection.	ADR: 29.1% vs. 20.3% (*p* < 0.001).	Prospective RCT.	Vendor and workflow dependencies.
Glissen Brown et al. (2022) [125]	CNN/Tandem colonoscopy.	Adenoma miss rate reduction.	AMR: 20.12% vs. 31.25% (OR 1.80, *p* = 0.025).	Prospective multicenter tandem RCT.	Tandem-colonoscopy design.
Wallace et al. (2022) [127]	Deep learning/Video.	Neoplasia miss rate reduction.	AMR: 15.5% vs. 32.4% (adjusted OR 0.38, *p* < 0.001).	Prospective tandem RCT.	Variable endoscopist experience.
Ishiyama et al. (2022) [129]	Propensity matched/Clinical.	Routine practice ADR sustainability.	ADR: 26.4% vs. 19.9% (RR 1.32, *p* = 0.001).	Prospective propensity-matched.	Propensity-matched single-center design.
Biscaglia et al. (2022) [130]	Real-time CADe/Endoscopy.	Trainee vs expert equalization.	Trainee ADR: 38% vs. Expert: 40% (not significant).	Prospective clinical.	Small trainee cohort size.
Nakashima et al. (2023) [133]	Novel AI/Endoscopy.	Neoplasia detection RCT.	ADR: 59.4% vs. 47.6% (*p* = 0.018).	Single-center RCT.	Single-center validation.
Gong et al. (2020) [7]	ENDOANGEL/Video.	Real-time adenoma detection.	ADR: 16% vs. 8% (OR 2.30, *p* = 0.001).	Single-center RCT.	Single-center validation.

### 5.2. Computer-Aided Diagnosis and Optical Biopsy

Computer-aided diagnosis systems (refer to Table 14) subsequently classify the detected lesions. These models differentiate adenomatous from hyperplastic histology by analyzing vascular and surface pit patterns. High-confidence optical diagnoses allow endoscopists to apply leave-in situ strategies for diminutive rectosigmoid polyps and resect-and-discard strategies for small adenomas, thereby reducing pathology costs and procedural time. Byrne et al. demonstrated that a deep learning model can differentiate adenomatous and hyperplastic diminutive colorectal polyps in real time using unaltered videos of standard colonoscopy [134]. Barua et al. validated real-time artificial intelligence-based optical diagnosis of neoplastic polyps during colonoscopy in a rigorous clinical evaluation [135]. Hassan et al. compared the performance of multiple artificial intelligence optical diagnosis systems and confirmed their suitability for leaving diminutive colorectal polyps in situ [119]. Oleksiw et al. established a framework for diagnostic agreement between the algorithm and the endoscopist, proving that high concordance rates support clinical implementation [17].

Advanced imaging modalities have better characterization accuracy. Teramoto et al. reviewed the updates in narrow-band imaging and demonstrated the integration of artificial intelligence with successive generations of optical filters to improve diagnostic accuracy [121]. Tamura et al. compared white light, magnifying endoscopy, endocytoscopy and artificial intelligence and concluded that algorithmic assistance has a similar diagnostic yield to ultrahigh magnification endocytoscopy without the need for specialized hardware [136]. Dos Santos et al. evaluated the performance of these models for characterizing different colorectal lesions and reported high specificity for malignant transformations [120]. A novel integrated computer-assisted system that combines detection and characterization into a single workflow, thereby simplifying the procedural interface, was validated by Weigt et al. [137]. Yoshida et al. analyzed the clinical practice of the CAD EYE system and confirmed that it can reliably recognize and diagnose lesions [138]. Van der Zander et al. compared a new computer-aided diagnosis system with intuitive optical diagnosis, showing enhanced algorithmic performance in novice practitioners [139].

### 5.3. Invasion Depth Prediction and T-Staging

Determining the depth of submucosal invasion in early colorectal cancer dictates the choice between endoscopic mucosal resection and radical surgical resection with lymph node dissection. Superficial submucosal invasion carries a low risk of lymph node metastasis, making endoscopic resection curative. Deep invasion necessitates surgery. Artificial intelligence models analyze magnifying narrow-band imaging, endocytoscopy, and non-magnified white-light images to predict invasion depth [140,141,142,143,144]. Whereas Nemoto et al. [140] provide a narrative synthesis of these approaches, the numerical values reported in Table 15 are drawn from the cited primary studies (Nakajima et al. [142]; Ichimasa et al. [141]; Kudo et al. [143]; Mori et al. [144]) rather than the narrative review itself. Nakajima et al. evaluated the diagnostic performance of artificial intelligence to identify deeply invasive colorectal cancer on non-magnified plain endoscopic images, proving that high-resolution magnification is not strictly required for accurate depth estimation [142]. Deep learning algorithms identify subtle morphological changes in the vascular network and surface structure that correlate with deep submucosal invasion. These models achieve comparable diagnostic accuracy to expert endoscopists and significantly outperform novice practitioners [141]. Mori et al. reviewed the clinical application of these models, concluding that algorithmic depth prediction reliably stratifies patients for endoscopic versus surgical management [144]. Endocytoscopy and AI-assisted endoscopic systems provide additional diagnostic information for evaluating deep submucosal invasion beyond standard magnifying chromoendoscopy [143,144]. Mitsala et al. and Liang et al. independently reviewed the broader landscape of artificial intelligence in T-staging, noting that algorithmic assistance standardizes the assessment of tumor depth across varying endoscopist skill levels [25,145].

### 5.4. Radiogenomics and Computational Pathomics

Molecular profiling is traditionally performed with targeted tissue sequencing, which is time-consuming and uses up limited biopsy material. Radiogenomics and computational pathomics derive molecular phenotypes directly from routine cross-sectional imaging and whole-slide images stained with hematoxylin and eosin. Deep learning models (refer to Table 16) identify subvisual spatial features that correlate with specific genomic alterations. Wang et al. showed that artificial intelligence can reliably detect colorectal cancer on histopathology slides. External patch-level validation was performed on NCT-CRC-HE-100K and CRC-VAL-HE-7K datasets containing 100,000 and 7180 patches, respectively, and AUC values of 98.32% and 98.45% were reported. In a separate clinical biopsy validation, the model demonstrated 100% sensitivity and high specificity for tumor detection in ileocolonoscopic biopsies across six external case cohorts digitized by four different scanners [8]. Tasi et al. used multi-omics machine learning to discover new colorectal cancer biomarkers, bridging the gap between genomic data and histological presentation [146]. Reitsam et al. surveyed the path of computational pathology, highlighting that these frameworks now directly personalize treatment decisions and discover novel biomarkers from resection slides [10]. Shimada et al. specifically validated histopathological characteristics and artificial intelligence for prediction of high tumor mutational burden, a critical metric for immunotherapy eligibility [147]. Studies have shown that convolutional neural networks can predict the mutation status of KRAS and BRAF from computed tomography radiomics with moderate accuracy [148].

Recent advancements utilize foundation models and hybrid architectures. Zhao et al. utilized multiview deep learning-enabled histopathology for prognostic and therapeutic stratification in stage II colorectal cancer, capturing spatial immune contexture across multiple magnification levels [149]. Qu et al. deployed a foundation model-enabled multimodal deep learning framework using whole-slide images, CT scans, and clinical reports to predict prognosis even when some modalities are missing [150]. Nallamalla et al. introduced a hybrid loss-based deep learning framework using EfficientNet-B3 for multi-class colorectal histopathology classification, improving class imbalance handling [151]. Hacking et al. validated whole-slide imaging for tumor budding and stromal differentiation, proving that algorithmic quantification reduces inter-observer variability in high-risk feature assessment [9]. Floares et al. introduced a circulating microRNA-based multi-cancer detection test utilizing generative and explainable artificial intelligence to map liquid biopsy signatures to tissue pathology [152].

**Table 16:** Summary of computational pathology and radiogenomics studies.

Study (Year)	Architecture/Modality	Clinical Task	Best Performance	Principal Limitation
Wang et al. (2021) [8]	CNN/H&E WSI.	CRC detection and biopsy tumor classification.	AUC: 98.32%, 98.45%; Sens: 100%, Spec: 96.74%.	Scanner, stain, and rare-class limitations.
Shimada et al. (2021) [147]	Deep learning/H&E.	Tumor mutational burden prediction.	AUC: 0.910 (TIL count) or 0.981 (CNN model).	Retrospective design.
Gonzalez et al. (2020) [148]	Radiomics/CT.	KRAS mutation detection.	Accuracy: 83%, Sens: 88.9%, Spec: 75.0%, Kappa: 64.7%.	Limited by CT spatial resolution.
Zhao et al. (2026) [149]	Multiview DL/Pathology.	Stage II prognostic stratification.	C-index: 0.74 for overall survival.	Requires multi-magnification scans.
Qu et al. (2026) [150]	Foundation model/Multimodal.	Prognosis with incomplete data.	C-index: 0.71 (multimodal fusion).	High computational overhead.
Hacking et al. (2020) [9]	CNN/WSI.	Tumor budding and stromal differentiation.	ICC: 0.89 for tumor budding quantification.	Requires standardized slide review.
Floares et al. (2024) [152]	Generative AI/miRNA.	Liquid biopsy to tissue mapping.	AUC: 0.91 for multi-cancer detection.	Requires complex multi-omics integration.
Nallamalla et al. (2026) [151]	EfficientNet-B3/Hybrid.	Multi-class cancer detection.	Accuracy: 94.2%; F1-score: 0.93.	Requires large GPU memory.
Cui et al. (2020) [153]	Radiomics/MRI.	KRAS mutation prediction.	AUC: 0.81 for KRAS status.	Limited by small cohort size.
Meng et al. (2019) [154]	Radiomics/Multiparametric MRI.	Biological characteristics (lymph node status, EMVI).	AUC: 0.85 for biological characteristics.	Requires multiparametric acquisition protocol.

H&E = hematoxylin and eosin. WSI = whole-slide image. CT = computed tomography. DL = deep learning.

### 5.5. Cross-Sectional Imaging and Metastasis Forecasting

Accurate staging and prognostic stratification demand the identification of distant metastases and the prediction of future recurrence. Ameyaw et al. reviewed artificial intelligence in colorectal imaging. They highlighted applications and opportunities for radiologists to improve cross-sectional staging [155], including algorithms using deep learning for detection of small hepatic metastases on contrast-enhanced computed tomography, which are often missed by human readers (refer to Table 17). Sartoris et al. developed a deep learning algorithm for liver metastasis detection at contrast-enhanced abdominal computed tomography and demonstrated superior sensitivity compared to independent radiologist reads [11]. Regarding curative resection, radiomics models based on the texture of the primary tumor and surrounding liver parenchyma can predict the appearance of metachronous liver metastases. Taghavi et al. validated a machine learning radiomics workflow with Bayesian hyperparameter optimization and five-fold cross-validation using clinical variables such as age, sex, primary tumor site, T-stage, N-stage and CEA [12]. Getu et al. developed machine learning and deep learning models for prediction of colorectal cancer metastasis in anatomical sites. They found 218 records from PubMed, SCOPUS, and Web of Science [156]. Rompianesi et al. focused on the diagnosis and management of liver metastases, stating how algorithmic volumetry and texture analysis guide surgical resection planning [13]. For instance, Wadhwani et al. proposed transformer-based multimodal fusion architectures that fuse lung and colorectal imaging data for the detection of synchronous metastatic disease [157]. Umehara et al. utilized deep ensemble learning for virtual endoluminal views in computed tomography colonography and improved polyp detection in three-dimensional reconstructed spaces [158].

### 5.6. Neoadjuvant Chemoradiotherapy Response Prediction

Locally advanced rectal cancer is commonly treated with neoadjuvant chemoradiotherapy before surgical resection. A subset of patients achieve a pathological complete response, and, for these individuals, organ-preserving strategies such as watch-and-wait surveillance may be considered. Accurate prediction of treatment response before or early during therapy is therefore a high-value clinical task. Artificial intelligence models that analyze baseline and mid-treatment imaging, histopathology, and clinical variables have been developed to predict pathological complete response and guide treatment de-escalation decisions.

Magnetic resonance imaging is the primary modality for rectal cancer staging and treatment response assessment. Radiomic models extract textural features from baseline and mid-treatment T2-weighted and diffusion-weighted sequences to predict tumor down-staging. Ferrari et al. developed an MR-based artificial intelligence model to assess response to therapy in locally advanced rectal cancer, demonstrating that quantitative imaging features extracted from pre-treatment scans correlate with pathological response categories [159]. Shi et al. extended this paradigm by applying machine learning to pre-treatment and mid-radiation multiparametric MRI for prediction of chemoradiation therapy response, showing that mid-treatment imaging features improve predictive accuracy over baseline features alone [160].

Deep learning approaches that operate directly on imaging volumes or pathology slides have also been explored. These models learn hierarchical features that capture subtle changes in tumor texture, cellularity, and stromal composition associated with treatment effect. Multimodal frameworks that integrate imaging, clinical, and molecular data are expected to further improve response prediction accuracy. Table 18 summarizes the principal artificial intelligence studies for neoadjuvant chemoradiotherapy response prediction in rectal cancer.

### 5.7. Surgical Phase Recognition and Human–Artificial Intelligence Interaction

Beyond diagnostics, the deployment of artificial intelligence in clinical workflows (refer to Table 19) encompasses surgical execution and human–computer operational deployment. Quero et al. investigated the present and future outlook of artificial intelligence in colorectal cancer surgery in the operating room, pointing out its utility in preoperative planning and intraoperative navigation [161]. An artificial intelligence-powered systematic review of colorectal surgery was conducted by Spinelli et al. confirming the improvement of surgical video analysis by algorithmic phase recognition and tool segmentation [26]. Kitaguchi et al. showed that deep learning models such as Xception and U-Net can be used to accurately perform phase recognition, action classification, and tool segmentation during laparoscopy [14]. Maier-Hein et al. contextualized these advances within surgical data science, outlining the path from algorithmic ideas to clinical translation [15].

To avoid automation bias, human–artificial intelligence interaction should be calibrated appropriately. van der Zander et al. investigated the suitable trust in artificial intelligence for the optical diagnosis of colorectal polyps, pointing out the necessity for endoscopists to adjust their trust in algorithmic outputs [16]. Reverberi et al. [162] experimentally demonstrated that humans and artificial intelligence can work together effectively in medical decision-making and that the combined performance is superior to the performance of the human or the algorithm alone. Besides the procedural interface, medical machine learning operations offer a framework for enabling the development and deployment of clinical artificial intelligence in radiology and endoscopy [18].

## 6. Datasets, Benchmarks, and Evaluation Standards

Public datasets, challenge benchmarks, and standardized evaluation metrics are essential for reproducible artificial intelligence in colorectal diagnosis. The field uses three broad categories of evidence resources: histopathology benchmarks, endoscopy benchmarks, and surgical workflow benchmarks. In addition, prospective clinical trials provide practical evaluation through clinical endpoints such as adenoma detection rate and adenomas per colonoscopy.

The histopathology datasets include large patch-level collections that are used for tissue classification and tumor detection (refer to Table 20). Wang et al. used external patch-level datasets, including NCT-CRC-HE-100K and CRC-VAL-HE-7K, to validate a colorectal cancer detection model across multiple scanners and pathology departments [8]. The CRAG (Colorectal Adenocarcinoma Gland) histology dataset was originally released alongside the Wang et al. [8] CRC detection model and is used for gland segmentation and tissue classification, while the SemiCOL challenge [163] extends multiclass colorectal tissue segmentation and weakly labeled resection specimen data using similarity-guided semi-supervised learning. The endoscopy benchmarks include polyp detection and segmentation datasets such as CVC-ClinicDB and Kvasir-SEG, which support algorithm comparison for polyp localization and segmentation [164,165]. The surgical workflow benchmarks include robotic instrument and scene segmentation challenges that are relevant to phase recognition, tool segmentation, and intraoperative assistance [15,166,167].

Evaluation should include task-appropriate metrics. Classification tasks use area under the receiver operating characteristic curve, sensitivity, specificity, accuracy, and calibration. Segmentation tasks use Dice similarity coefficient, intersection over union, and boundary metrics. Clinical endoscopy studies use adenoma detection rate, adenomas per colonoscopy, miss rate, and procedural time. For clinical translation, internal validation is not enough. Temporal validation, external validation, prospective evaluation, and human–artificial intelligence interaction studies are necessary [16,17,18]. Reporting should follow the frameworks summarized in Table 12.

### Data Leakage, Patient Overlap, and Benchmark Contamination

The combined evidence in this review has several risks of analytical bias. Patient-level overlap: If training and test data come from the same institution, patient-level leakage can inflate performance. Studies that are separated by image or patch, not by patient identity, are particularly vulnerable. Splitting at the patient level was not explicitly reported in most of the included studies. Readers should be wary of per-patch or per-image metrics unless patient-level grouping can be confirmed. Benchmark contamination: Training and testing are performed on popular public datasets, such as NCT-CRC-HE-100K, CRC-VAL-HE-7K, CVC-ClinicDB, and Kvasir-SEG. When a model that was trained on one public dataset is evaluated on another dataset that contains source images or source patients, the reported performance and apparent generalizability may be overstated. While Wang et al. [8] addressed this issue by training on NCT-CRC-HE-100K and externally validating on CRC-VAL-HE-7K, not every study reported such rigorous separation.

Temporal and distributional shift: Models that are trained on retrospective data of a fixed time window may not generalize to prospective populations with different disease prevalence, bowel preparation quality and imaging hardware. This concern has been directly addressed only by prospective CADe trials [5,6,7,125]. Recommendation: Future studies should (i) report the splitting unit (patient, lesion, image or patch), (ii) declare any overlap between training and evaluation sources, (iii) include at least one external cohort from a different institution and scanner, and (iv) report performance stratified by subgroups that are relevant to distributional shift (e.g., bowel preparation quality, polyp size, and histological subtype).

## 7. Open Challenges and Future Directions

There are many barriers to the clinical use of artificial intelligence in colorectal diagnosis (refer to Table 21). Established Evidence: Computer-Aided Detection and Optical Diagnosis: CADe has been shown to improve the adenoma detection rate and reduce the adenoma miss rate in several randomized controlled trials and large prospective studies [5,6,7,125,127]. Optical diagnosis systems have been validated for leave-in situ strategies with high concordance versus histopathology [119,134,135]. These are the most mature applications and are the closest to routine clinical use. The challenges that remain in this tier include the standardization of reporting of adverse device events across vendors, long-term surveillance data for resect-and-discard strategies, and alignment of reimbursement.

Validated but Not Yet Deployed: Computational Pathology, Radiogenomics, and Metastasis Prediction: Deep learning models for histopathology classification [8], tumor budding quantification [9], and radiomics metastasis prediction [12] have shown strong retrospective performance with external validation. However, none has undergone sufficient prospective multi-site validation for regulatory submission. Exploratory Evidence: Microbiome, VOC, Nanotechnology, and Surgical Workflow: Microbiome classifiers [32], VOC-based screening, nanotechnology-enhanced biosensors [107], and surgical phase recognition [14] are in early stages. The sample sizes are small, external validation is lacking or limited, and clinical utility has not been demonstrated. Prospective cohort studies are needed in these areas to make translational claims.

Cross-Cutting Barriers: There are barriers to clinical adoption of artificial intelligence across all evidence tiers. First, domain shift is still prevalent. Differences in the scanner, variation in the stain, quality of the bowel preparation, lighting, and vendor-specific video pipelines can degrade performance. Hence, external validation across institutions is necessary [8,151]. Second is the importance of calibration and trust. Clinicians need to know when to trust a model and when to override a model. Appropriate trust, uncertainty quantification, and explanation stability are important for safe human–artificial intelligence collaboration [16,17,162]. Third, multimodal foundation models bring new opportunities and risks. Models that integrate pathology, imaging, clinical text, and molecular data may improve prognostic inference, but missing modalities, high computational cost, and opaque attention need careful evaluation [149,150].

Future Work: Five research priorities emerge from this review. First, prospective multicenter randomized controlled trials are needed to validate computational pathology and radiogenomics models under real-world clinical conditions, including particular attention to domain shift across scanners, stains, and patient populations. Second, standardized benchmarking protocols must be established for emerging modalities, including microbiome classifiers and nanotechnology biosensors, to enable reproducible cross-study comparison. Third, multimodal foundation models that integrate pathology, imaging, clinical text, and molecular data require rigorous evaluation of their robustness to missing modalities, computational cost, and attention interpretability before clinical deployment. Fourth, the field must address the pervasive risk of patient-level data leakage and benchmark contamination by mandating transparent reporting of splitting units, training-evaluation overlap, and external cohort provenance. Fifth, human–artificial intelligence interaction studies should be expanded beyond endoscopy to include pathology, radiology, and multidisciplinary tumor board settings with calibrated trust metrics and automation bias assessments.

Clinical Implementation Pathway: Translating validated colorectal artificial intelligence models from research to routine clinical practice requires a structured pathway. The first stage involves retrospective external validation on diverse multi-institutional cohorts with explicit documentation of domain shift mitigation strategies. The second stage requires prospective clinical utility studies, ideally randomized controlled trials, that demonstrate improved patient outcomes or workflow efficiency rather than standalone diagnostic accuracy. The third stage involves regulatory submission through the appropriate jurisdictional pathway, such as the FDA 510(k) [20] or De Novo classification [21] for the United States or the EU MDR conformity assessment for European deployment [22]. The fourth stage encompasses post-deployment monitoring through machine learning operations frameworks, including continuous drift detection, periodic recalibration, and adverse event reporting. Finally, sustainable clinical integration demands alignment of reimbursement structures, clinician training programs, and interoperability standards to ensure that validated artificial intelligence tools are adopted equitably across diverse healthcare settings. For clinically useful artificial intelligence at all maturity tiers, robust external validation, uncertainty quantification, explainable outputs, calibration testing, and standardized reporting per TRIPOD-AI, CONSORT-AI, or CLAIM as appropriate remain essential prerequisites.

### Regulatory Pathways for Colorectal AI Devices

Translating a validated colorectal AI model into a clinically deployed device includes compliance with jurisdiction-specific regulations. The following walkthrough demonstrates the FDA pathway for a hypothetical endoscopic AI device; the European Union Medical Device Regulation (EU MDR 2017/745) follows similar but different steps. FDA Pathway: 510(k) vs. De Novo: An endoscopic AI tool that assists gastroenterologists in detecting or characterizing colorectal polyps during colonoscopy would be classified as a computer-aided detection/diagnosis (CADe/CADx) device. For example, the Medtronic GI Genius system successfully achieved FDA De Novo clearance as a CADe device [24]. Where a legally marketed predicate device exists with the same intended use and technological characteristics, the manufacturer may submit a 510(k) premarket notification demonstrating substantial equivalence. If the existing predicates cannot be applied, as is often the case for new multimodal or foundation model-based tools, the De Novo classification pathway is needed. The De Novo process creates a new device classification (Class II for most colorectal AI tools) and a new regulation for that classification. The key elements of a submission for a colorectal AI device are summarized in Table 22.

EU MDR Classification: According to EU MDR 2017/745, a colorectal AI diagnostic tool would typically fall under Rule 11 of Annex VIII (software intended to supply information to be used for diagnostic or therapeutic decisions). A decision-impacting tool with significant consequences (e.g., predicting invasion depth that determines endoscopic resection or surgical referral) would be classified as Class IIa or IIb, requiring a conformity assessment by a Notified Body, a clinical evaluation report, and post-market surveillance including periodic safety update reports. The above regulatory walkthrough links the abstract phases of the clinical translation pipeline to actionable requirements. Developers of colorectal AI tools should engage regulatory consultants early in the development cycle to align study design with submission expectations.

## 8. Conclusions

This review synthesized 160 references across nine diagnostic modalities and seven task-specific artificial intelligence domains to provide a comprehensive assessment of the current state of artificial intelligence for colorectal cancer diagnosis. The evidence reveals a wide maturity gradient. Computer-aided detection and optical diagnosis in endoscopy are the most advanced applications, supported by Level 1 evidence from multiple randomized controlled trials demonstrating sustained improvements in adenoma detection rates and miss rate reductions. These systems are the closest to routine clinical deployment, although challenges remain in vendor standardization, long-term surveillance outcomes, and reimbursement alignment. Computational pathology, radiogenomics, and metastasis prediction occupy an intermediate tier: deep learning models for histopathology classification, tumor budding quantification, and radiomics-based metastasis forecasting have demonstrated strong retrospective performance with external validation, but none has yet undergone the required prospective multi-site validation for regulatory submission. At the exploratory end of the spectrum, microbiome-based screening, volatile organic compound profiling, nanotechnology-enhanced biosensors, and surgical phase recognition remain in early-stage development with small sample sizes, limited external validation, and no demonstrated clinical utility in prospective cohorts.

## Figures and Tables

**Table 1:** Inclusion and exclusion criteria applied during study selection.

Inclusion Criteria	Exclusion Criteria
Studies reporting artificial intelligence, machine learning, or computer-aided diagnosis methods for colorectal disease detection, characterization, staging, prognosis, or workflow integration.	Studies limited to manual diagnosis without artificial intelligence integration.
Studies using endoscopy, radiology, histopathology, molecular diagnostics, liquid biopsy, or multimodal colorectal data.	Non-colorectal studies without direct relevance to colorectal diagnosis.
Articles with quantitative evaluation, validation metrics, or structured reporting that is relevant to clinical translation.	Records without quantitative results, inaccessible full texts, or non-English publications.

**Table 2:** Structured evidence grading by clinical task.

Clinical Task	Evidence Level	Consistency	Clinical Readiness	No. of Studies
CADe in endoscopy	1	High	Validated/Deployed	8
Optical diagnosis (CADx)	1	High	Validated	8
Invasion depth prediction	2	Moderate	Exploratory	6
Radiogenomics/pathomics	2	Moderate	Validated	10
Metastasis forecasting	2–3	Moderate	Exploratory	6
nCRT response prediction	2–3	Low	Exploratory	2
Surgical phase recognition	2–3	Moderate	Exploratory	7
Microbiome/VOC screening	3	Low	Exploratory	3
Nanotechnology diagnostics	3	Low	Preclinical	4

Evidence Level: 1 = prospective/RCT with external validation; 2 = retrospective with internal or external validation; 3 = single-site, preclinical, or case series. Consistency and clinical readiness are defined in Section 2.4.

**Table 3:** Differentiation of the proposed survey from existing studies on artificial intelligence in colorectal disease. Last column (in red) refers to the current study.

Feature or Scope	Liang et al. [25]	Reitsam et al. [10]	Spinelli et al. [26]	Nazarian et al. [27]	This Survey
Endoscopic CADe and CADx	∼	✓	✕	✓	✓
Cross-sectional imaging and radiomics	∼	∼	∼	✕	✓
Computational pathology and MIL	∼	∼	∼	✕	✓
Molecular, liquid biopsy, and multi-omics AI	∼	∼	✕	✕	✓
Neoadjuvant treatment response prediction	∼	∼	∼	✕	✓
Surgical phase recognition and workflow	∼	∼	✓	✕	✓
Multimodal fusion and NLP or LLM integration	∼	✕	✕	✕	✓
Nanotechnology-based diagnostics	✕	✕	✕	✕	✓
AI reporting standards and governance	✕	✕	✕	✕	✓
Dataset and benchmark catalog	✕	✕	✕	✕	✓

✓ = comprehensively covered (dedicated section or extensive analysis). ∼ = partially covered or limited scope (brief mention or narrow focus). ✕ = not covered (absent from the review).

**Table 4:** Clinical risk stratification and recommended diagnostic pathways in colorectal cancer screening.

Risk Category	Defining Features	Recommended Pathway	Screening Interval
Average risk	Age 45 to 75, no family history, no hereditary syndrome.	Stool-based test or colonoscopy.	Annual FIT or 10-year colonoscopy.
Increased risk	First-degree relative with CRC or advanced adenoma before age 60.	Colonoscopy preferred.	Every 5 years starting at age 40 or 10 years before youngest affected relative.
High risk (Lynch syndrome)	Lynch syndrome.	Colonoscopy with chromoendoscopy or high-definition imaging.	Every 1 to 2 years starting at age 20 to 25 [30].
High risk (FAP)	Familial adenomatous polyposis.	Colonoscopy or sigmoidoscopy.	Every 1 to 2 years starting at age 10 to 15 [30].
High risk (IBD)	Inflammatory bowel disease.	Surveillance chromoendoscopy or high-definition WLI.	Every 1 to 3 years starting 8 years after diagnosis [3].
Symptomatic	Rectal bleeding, iron-deficiency anemia, unexplained weight loss, or change in bowel habits.	Diagnostic colonoscopy with or without cross-sectional imaging.	Immediate evaluation.

CRC = colorectal cancer. FIT = fecal immunochemical test.

**Table 5:** Stool-based screening tests for colorectal cancer.

Test	Mechanism	Analytical Target	Sens. (Reported Target)	Interval	Cost	Key Limitation
gFOBT	Heme pseudoperoxidase activity on guaiac paper.	Occult hemoglobin (non-specific).	12%	Annual	Low	Dietary interference, low adenoma sensitivity [1,35].
FIT	Monoclonal or polyclonal antibodies targeting human globin.	Human globin (colon-specific).	79%	Annual	Moderate	Misses advanced adenomas [36,37,38,39].
mt-sDNA	FIT combined with tumor-associated DNA biomarkers.	Hemoglobin, KRAS, BMP3, NDRG4, beta-actin.	92%	3 years	High	High false-positive rate, costly [2,40,41].

Sens. = sensitivity for the reported target. The gFOBT value refers to carcinoma detection (per Zavoral et al. [35]; the corresponding advanced-adenoma sensitivity is 11%). The FIT value refers to colorectal cancer detection (pooled sensitivity 79% and specificity 94% per Lee et al. [37]); the corresponding advanced-adenoma sensitivity is approximately 25%. The mt-sDNA value refers to colorectal cancer detection (sensitivity 92.3% per Imperiale et al. [41]). Cost tiers are qualitative and intended for relative comparison.

**Table 6:** Imaging modalities for colorectal cancer evaluation.

Modality	Primary Role	Radiation	Sedation	Soft-Tissue Contrast	Availability	Key Limitation
CT colonography	Alternative screening when colonoscopy is contraindicated.	✓	✕	∼	∼	Requires full bowel preparation [42,45,46].
MRI	Local rectal cancer staging.	✕	✕	✓	∼	Long scan time, implant restrictions [47,48,49,50].
PET/CT	Recurrence detection and metastasis survey.	✓	✕	∼	✕	Low spatial resolution, high cost [52,53,54,55].
Ultrasound	Local staging via RUS and CE-EUS.	✕	✕	∼	✓	Operator-dependent, limited deep tissue access [54,60,61,62,63].

CT colonography = computed tomography colonography. RUS = rectal ultrasound. CE-EUS = contrast-enhanced endoscopic ultrasound. ✓ = present or high. ∼ = moderate or variable. ✕ = absent or limited.

**Table 7:** Endoscopic modalities for direct visualization.

Modality	Anatomical Reach	Biopsy	Sedation	Full Prep	Interval	Key Limitation
Colonoscopy	Entire colon and terminal ileum.	✓	✓	✓	10 years	Perforation risk, resource-intensive [31,64,65,67,68].
Flexible sigmoidoscopy	Rectum, sigmoid, descending colon.	✓	✕	∼	5 years	Misses proximal lesions entirely [29,69,70].
Colon capsule endoscopy (CCE)	Entire colon via peristalsis.	✕	✕	✓	Not established	No therapeutic capability, incomplete transit [3,71,72,73,74].

Biopsy = tissue sampling and polypectomy capability. Sedation = intravenous sedation typically required. Full Prep = cathartic bowel preparation required. Interval = recommended screening interval for average-risk adults. ✓ = yes. ∼ = partial (enema only). ✕ = no.

**Table 8:** Comprehensive comparison of endoscopic and imaging modalities for colorectal cancer diagnosis and staging.

Criterion	Colonoscopy	Sigmoidoscopy	Colon Capsule Endoscopy	CT Colonography	MRI	PET/CT	Ultrasound (ERUS/TAUS)
Cost	High	Low–Moderate	Moderate–High	Moderate–High	High	Very High	Low
Scan Time	15–45 min	10–20 min	8–12 h recording	10–20 min	30–60 min	3 h	10–30 min
Invasiveness	High	Moderate	Minimally invasive	Minimally invasive	Non-invasive	Minimally invasive (IV tracer)	Non-invasive
Radiation	None	None	None	Yes	None	Yes	None
Clinical Indication	Gold standard for CRC detection, biopsy, and polypectomy.	Distal colon and rectal screening.	CRC screening when colonoscopy is incomplete or refused.	CRC screening when colonoscopy is incomplete or contraindicated.	Local staging of rectal cancer.	Detection of recurrence, metastasis, and treatment response.	Local staging of rectal cancer and liver metastasis evaluation.
Cancer Compatibility	Excellent for primary CRC and precancerous lesions.	Limited to left-sided CRC.	Good for detecting polyps; no biopsy capability.	Good for large polyps and cancers.	Excellent for rectal cancer staging.	Excellent for recurrent/metastatic CRC; limited for primary staging.	Good for early rectal cancer; limited for distant disease.
False Positive Rate	Low	Moderate	Moderate	Low–Moderate	Low	Moderate (inflammation)	Moderate
False Negative Rate	Low (may miss flat lesions)	Higher (misses proximal lesions)	Moderate (incomplete transit)	Higher for small (<6 mm) and flat lesions	Moderate (early T-stage)	Moderate (small/mucinous tumors)	Moderate (operator-dependent)
Supporting Citations	[31,64,65,67,68]	[29,69,70]	[3,71,72,73,74]	[42,45,46]	[47,48,49,50]	[52,53,54,55]	[54,60,61,62,63]

CRC = colorectal cancer. ERUS = endorectal ultrasound. TAUS = transabdominal ultrasound. IV = intravenous. Cost categories are qualitative and intended for relative comparison.

**Table 9:** Histopathological processing and evaluation parameters.

Stage	Key Actions	Clinical Significance	AI-Ready	Automation
Gross Examination	Margin inking, specimen dissection, mesorectal assessment.	Determines serosal involvement and circumferential margin status [76,77]	∼	✕
Tissue Processing	Formalin fixation, dehydration, paraffin embedding, microtome sectioning.	Preserves morphology for downstream staining and analysis [77]	✓	✓
H&E Staining	Staining and microscopic evaluation of architecture and cellular morphology.	Confirms malignancy and determines tumor grade [4,75,78]	✓	∼
Feature Assessment	Margin, lymphovascular invasion, perineural invasion, tumor budding.	Dictates adjuvant therapy selection and prognostic stratification [4,78]	✓	∼

AI-Ready = digitized whole-slide image available for computational analysis. Automation = current level of laboratory automation for this stage. ✓ = high. ∼ = partial or emerging. ✕ = manual only.

**Table 10:** Molecular diagnostics and biomarker categories.

Category	Key Targets	Sample Type	Validation	Readiness	Clinical Utility
Blood-Based Biomarkers	CEA, CA19-9.	Serum.	✓	Routine	Prognosis and recurrence monitoring [42,79].
Liquid Biopsy	CTCs, cfDNA, exosomes.	Peripheral blood.	∼	Emerging	Non-invasive real-time disease tracking [80,81,82,84].
Genomic	Clinically actionable targets include KRAS and BRAF. Research and prognostic targets include APC, TP53, PIK3CA, and SMAD4.	Tissue, blood.	✓	Routine for actionable targets	Guides targeted therapy and resistance assessment; APC and TP53 are primarily research or prognostic markers [85,86,87,88,89,90,91,92,93,94].
Proteomic	Protein expression profiles.	Tissue, blood.	✕	Research	Biomarker discovery and target identification [101,105].
Epigenetic	SEPT9, NDRG4, BMP3 methylation.	Blood, stool.	∼	Emerging	Early detection in blood and stool assays [40,95].
Transcriptomic	miRNAs, lncRNAs, circRNAs.	Blood, tissue.	✕	Research	Minimally invasive expression profiling [96,97,98,100].
Metabolomic	Small-molecule metabolites.	Blood, urine.	✕	Research	Captures tumor metabolic reprogramming [102,103,104].

CTC = circulating tumor cell. cfDNA = cell-free DNA. Validation = large-scale clinical validation completed. Readiness = current clinical implementation status. ✓ = established. ∼ = partial. ✕ = not yet validated.

**Table 11:** Nanotechnology platforms for colorectal diagnostics.

Class	Composition	Application	Biocompat.	Maturity	Translational Barrier
Quantum Dots	Fluorescent semiconductor nanocrystals.	CTC detection, molecular imaging.	✕	Preclinical	Toxicity concerns, limited in vivo validation [106,107,108].
Carbon-Based	Nanotubes, graphene, carbon dots.	Biosensors, sentinel lymph node mapping.	∼	Early Clinical	Manufacturing scalability, long-term safety data [109,110].
Lipid-Based	Phospholipids, cholesterol.	Targeted contrast agent encapsulation.	✓	Early Clinical	Formulation stability, regulatory approval [111].
Gold-Based	Gold nanospheres, nanorods, and nanoshells.	Targeted molecular imaging, biosensing, and photothermal contrast enhancement.	✓	Preclinical	Standardized synthesis, biodistribution, and regulatory approval [106,107].

CTC = circulating tumor cell. QD = quantum dot. Biocompat. = biocompatibility rating based on current evidence. Maturity = current translational stage. ✓ = high. ∼ = moderate. ✕ = low or not established.

**Table 12:** Reporting and governance frameworks for artificial intelligence in colorectal diagnosis.

Framework	Primary Focus	Applicable Design	Imaging	XAI	Calib.	Ext. Val.	Section
TRIPOD-AI	Transparent reporting of prediction model development and validation.	Retrospective or prospective cohort, registry.	∼	∼	✓	✓	Methods, Results
CONSORT-AI	Reporting of randomized clinical trials evaluating artificial intelligence interventions.	Randomized controlled trial.	∼	✓	✓	✓	Methods, Results
SPIRIT-AI	Protocol-level reporting for artificial intelligence clinical trial design.	Trial protocol and pre-registration.	✕	✕	✕	✕	Methods
DECIDE-AI	Human-centered evaluation of decision support systems in clinical workflow.	Prospective usability and safety study.	✓	✓	∼	∼	Methods, Discussion
CLAIM	Structured reporting for medical imaging studies using artificial intelligence.	Retrospective or prospective imaging study.	✓	✓	✓	✓	Methods, Results

✓ = comprehensively addressed. ∼ = partially addressed or implied. ✕ = not addressed. XAI = explainable artificial intelligence. Calib. = calibration reporting. Ext. Val. = external validation requirement.

**Table 14:** Summary of computer-aided diagnosis and optical biopsy studies.

Study (Year)	Architecture/Modality	Clinical Task	Best Performance	Principal Limitation
Byrne et al. (2019) [134]	CNN/Standard video.	Adenoma vs. hyperplastic classification.	Accuracy: 94.0%; Sens: 98.0%; Spec: 83.0%.	Diminutive polyps only.
Barua et al. (2022) [135]	Real-time AI/Endoscopy.	Neoplastic polyp diagnosis.	CADx Sens: 90.4%; Spec: 85.9% ((not significant) vs. visual inspection).	Threshold-dependent performance.
Hassan et al. (2023) [119]	Multi-system comparison.	Leave-in situ suitability.	NPV: 97.8% for ≤5 mm rectosigmoid polyps; ≥90% surveillance concordance.	Variable lighting conditions.
Oleksiw et al. (2026) [17]	Agreement framework.	Endoscopist-AI concordance.	Agreement kappa: 0.82.	Requires baseline endoscopist data.
Tamura et al. (2026) [136]	AI vs Endocytoscopy.	Early CRC diagnosis.	AUC: 0.96; Accuracy: 93.0%.	Retrospective image analysis.
Weigt et al. (2022) [137]	Integrated CADe/CADx.	Detection and characterization.	CADe Sens: 92.9%, Spec: 90.6% (WLI); CADx Acc: 83.6%.	Vendor integration constraints.
Yoshida et al. (2021) [138]	CAD EYE/Clinical.	Lesion recognition and diagnosis.	CADx Accuracy: 88.8% (non-magnified BLI); CADe detection: 85.0% (WLI).	Single-vendor ecosystem.
van der Zander et al. (2021) [139]	Novel CADx/WLI.	Novice vs algorithm comparison.	CADx multimodal Acc: 95.0% vs. Novice: 66.7% (*p* < 0.001).	Limited to static image analysis.

CADe = computer-aided detection. CADx = computer-aided diagnosis. CRC = colorectal cancer. WLI = white-light imaging.

**Table 15:** Summary of artificial intelligence models for invasion depth prediction and T-staging.

Study (Year)	Architecture/Modality	Clinical Task	Best Performance	Principal Limitation
Nakajima et al. (2020) [142]	CNN/Non-magnified WL.	Deep invasion identification.	AUC: 0.89; Accuracy: 84%.	Non-magnified image setting.
Ichimasa et al. (2018) [141]	Machine learning/Endoscopy.	Need for additional surgery.	Sens: 100%; Spec: 66%; Acc: 69% for LNM prediction.	Small T1 cancer cohort.
Nemoto et al. (2026) [140]	Deep learning/Video.	Submucosal invasion depth.	Specificity: 94.4%; Sensitivity: 59.8% (narrative review; primary studies cited within).	Requires standardized video capture.
Mori et al. (2020) [144]	Systematic Review/AI.	Clinical application synthesis.	–	Heterogeneous study designs.
Kudo et al. (2014) [143]	Endocytoscopy/AI.	Deep submucosal invasion.	–	Requires specialized endocytoscope.
Mitsala et al. (2021) [145]	Review/T-staging.	Algorithmic T-staging standardization.	Kappa improved: 0.45 to 0.81.	Lack of prospective RCTs.

WL = white light. Values marked as “Pooled” are derived from published systematic reviews or meta-analyses; all other values are individual-study results reported in the primary literature. No values were calculated specifically for this review.

**Table 17:** Summary of cross-sectional imaging and metastasis forecasting studies.

Study (Year)	Architecture/Modality	Clinical Task	Best Performance	Principal Limitation
Sartoris et al. (2026) [11]	DL/Contrast CT.	Liver metastasis detection.	Per-nodule detection rate: 81% (vs R1: 79%, R2: 76%); false detection rate: 22%.	False positives in diseased livers.
Taghavi et al. (2021) [12]	ML/CT Radiomics.	Metachronous metastasis prediction.	AUC: 0.86 (95% CI 85-87%) for metachronous metastasis.	Requires long-term follow-up data.
Getu et al. (2026) [156]	Systematic Review/ML.	Multi-site metastasis forecasting.	Pooled AUC: 0.87 across reviewed models.	Heterogeneous evaluation metrics.
Rompianesi et al. (2022) [13]	AI/CT and MRI.	Liver metastasis management.	Volumetric error < 5%; AUC: 0.81.	Complex workflow integration.
Wadhwani et al. (2026) [157]	Transformers/Multimodal.	Synchronous metastasis detection.	AUC: 0.91; Sensitivity: 89.0%.	High computational memory demand.
Umehara et al. (2017) [158]	Deep ensemble/CT colonography.	Virtual endoluminal polyp detection.	Sensitivity: 95.0%; FP per scan: 1.2.	Dependent on CT colonography reconstruction quality.

ML = machine learning. MRI = magnetic resonance imaging. Values marked as “Pooled” are derived from published systematic reviews or meta-analyses; all other values are individual-study results reported in the primary literature. No values were calculated specifically for this review.

**Table 18:** Summary of artificial intelligence studies for neoadjuvant chemoradiotherapy response prediction in rectal cancer.

Study (Year)	Architecture/Modality	Clinical Task	Best Performance	Principal Limitation
Ferrari et al. (2019) [159]	AI/MRI.	Response assessment in locally advanced rectal cancer.	AUC: 0.86 for CR classification; AUC: 0.83 for NR classification.	Retrospective single-institution design.
Shi et al. (2019) [160]	Machine learning/Multiparametric MRI.	Chemoradiation response prediction using pre-treatment and mid-radiation features.	AUC: 0.82 (mid-treatment) vs 0.80 (baseline) for ROI+radiomics pCR prediction.	Requires standardized mid-treatment imaging protocol.

MRI = magnetic resonance imaging. KRAS = Kirsten rat sarcoma viral oncogene homolog. Values marked as “Pooled” are derived from published systematic reviews or meta-analyses; all other values are individual-study results reported in the primary literature. No values were calculated specifically for this review.

**Table 19:** Summary of surgical artificial intelligence and human–computer interaction studies.

Study (Year)	Architecture/Modality	Clinical Task	Best Performance	Principal Limitation
Kitaguchi et al. (2020) [14]	Xception, U-Net/Video.	Surgical phase and tool segmentation.	Phase accuracy: 91.9%; Extracorporeal action F1: 89%; Irrigation action F1: 80%.	Limited to specific laparoscopic views.
Spinelli et al. (2023) [26]	Systematic Review/Surgical AI.	Intraoperative navigation synthesis.	Pooled phase accuracy: 89.5%.	High risk of bias in included trials.
van der Zander et al. (2024) [16]	Human-AI interaction/CADx.	Appropriate trust calibration.	Calibration error reduced by 34%.	Requires extensive endoscopist training.
Reverberi et al. (2022) [162]	Experimental/Decision-making.	Collaborative performance.	S2 (hybrid) accuracy: 80.2% vs. S1 (human alone): 76.8%; AI alone: 84.9%.	Difficult to quantify in standard trials.
de Almeida et al. (2025) [18]	MLOps/Deployment.	Model monitoring and drift detection.	Drift detection latency < 24 h.	Requires dedicated data engineering.
Quero et al. (2022) [161]	Review/Surgical AI.	Preoperative and intraoperative utility.	Mapped 45 intraoperative use-cases.	Lack of prospective surgical RCTs.
Maier-Hein et al. (2022) [15]	Conceptual/Surgical Data Science.	Clinical translation pathway.	Defined 5-stage translation framework.	Theoretical rather than empirical.

MLOps = machine learning operations. CADx = computer-aided diagnosis. RCT = randomized controlled trial.

**Table 20:** Representative datasets, benchmarks, and evaluation resources for colorectal artificial intelligence.

Resource	Domain	Task	Principal Metrics	Strength and Limitation
NCT-CRC-HE-100K/CRC-VAL-HE-7K [8]	H&E histology patches.	Tissue classification and tumor detection.	Accuracy, AUC, sensitivity.	Large patch-level benchmark; limited outcome labels.
CRAG/SemiCOL [8,163]	Colorectal histology.	Multiclass tissue segmentation.	Dice, IoU.	Challenge-style annotations; gland and tissue-level labels.
CVC-ClinicDB/MICCAI polyp challenge [164]	Colonoscopy video.	Polyp detection and segmentation.	Sensitivity, specificity, Dice.	Challenge protocol; limited size.
Kvasir-SEG [165]	Colonoscopy images.	Polyp segmentation.	Dice, IoU.	Public segmentation masks; domain shift possible.
EndoVis and robotic instrument challenges [166,167]	Surgical video.	Instrument and scene segmentation.	Dice, accuracy.	Surgical AI benchmark; not colorectal-specific.
Heidelberg colorectal surgical dataset [168]	Surgical video.	Workflow recognition and phase segmentation.	Accuracy, Dice.	Sensor operating room annotations; colorectal-specific.
Prospective CADe trials [5,6,7,125]	Clinical colonoscopy.	Real-time polyp detection.	ADR, APC, miss rate.	Clinical endpoints; not public datasets.

H&E = hematoxylin and eosin. AUC = area under the receiver operating characteristic curve. IoU = intersection over union. ADR = adenoma detection rate. APC = adenomas per colonoscopy. CADe = computer-aided detection.

**Table 21:** Open challenges and recommended evaluation strategies for artificial intelligence in colorectal diagnosis.

Challenge	Driver	Recommended Action
Domain shift and stain variability	Scanner, stain, lighting, and vendor differences.	External multi-site validation, normalization, and scanner diversity [8,151].
Limited prospective evidence	Retrospective studies do not prove workflow benefit.	Randomized trials and prospective workflow studies [5,7,26].
Calibration and trust	Automation bias and overreliance.	Uncertainty quantification, explanation stability, and clinician-in-the-loop evaluation [16,17].
Multimodal missing data	Incomplete pathology, imaging, clinical, and molecular inputs.	Robust fusion, foundation models, and missing-modality testing [149,150].
Deployment and monitoring	Model drift and governance requirements.	MLOps, continuous monitoring, and regulatory alignment [15,18].

**Table 22:** Key elements of a regulatory submission for a colorectal AI device under the FDA pathway.

Submission Element	Description and Requirements
Intended use statement	Provides clinical context (e.g., “adjunctive tool for real-time detection of colorectal polyps ≥ 5 mm during screening or surveillance colonoscopy in adults aged 45 years or older”), intended user (gastroenterologist or endoscopist) and clinical setting.
Analytical validation	Technical performance on a locked representative dataset including sensitivity, specificity, AUC, and calibration across subgroups by polyp morphology, size, location, and quality of bowel preparation.
Clinical validation	Multi-reader multi-case (MRMC) reader studies showing improvement in adenoma detection rate or diagnostic accuracy with the AI tool compared to unassisted reading. To reduce domain shift, the study should involve at least three endoscopy centers and multiple video-capture systems.
Algorithm transparency	Description of model architecture, training data provenance, preprocessing pipeline and any post-market learning or update plan. The FDA Predetermined Change Control Plan framework is applicable when the algorithm will be updated after clearance [23].
Cybersecurity and data privacy	HIPAA compliance for US patient data, including de-identification processes, data-use agreements, and secure transmission protocols.

## Data Availability

No new data were created or analyzed in this study. The extracted dataset of 160 included studies, along with the list of full-text exclusions and the PRISMA 2020 checklist, is provided in the Supplementary Materials.

## Supplementary Materials

The following supporting information can be downloaded at: https://www.mdpi.com/article/10.3390/cancers18213522, Table S1: PRISMA 2020 checklist.

## Abbreviations

The following abbreviations are used in this manuscript:

Abbreviation	Definition
ADR	Adenoma detection rate
AI	Artificial intelligence
APC	Adenomas per colonoscopy; adenomatous polyposis coli
AUC	Area under the receiver operating characteristic curve
CA19-9	Carbohydrate antigen 19-9
CADe	Computer-aided detection
CADx	Computer-aided diagnosis
CE-EUS	Contrast-enhanced endoscopic ultrasound
CEA	Carcinoembryonic antigen
CCE	Colon capsule endoscopy
cfDNA	Cell-free DNA
CLAIM	Checklist for Artificial Intelligence in Medical Imaging
CNN	Convolutional neural network
CONSORT-AI	Consolidated Standards of Reporting Trials for Artificial Intelligence Interventions
CRC	Colorectal cancer
CT	Computed tomography
CTC	Circulating tumor cell
DECIDE-AI	Human-centered evaluation of decision support systems in clinical workflow
DICOM	Digital Imaging and Communications in Medicine
EMVI	Extramural vascular invasion
ERUS	Endorectal ultrasound
FIT	Fecal immunochemical test
GAN	Generative adversarial network
gFOBT	Guaiac fecal occult blood test
Grad-CAM	Gradient-weighted class activation mapping
H&E	Hematoxylin and eosin
IoU	Intersection over union
KRAS	Kirsten rat sarcoma viral oncogene homolog
LLM	Large language model
MIL	Multiple instance learning
MLOps	Machine learning operations
MRI	Magnetic resonance imaging
mt-sDNA	Multitarget stool DNA
NLP	Natural language processing
PET/CT	Positron emission tomography/computed tomography
PRISMA	Preferred Reporting Items for Systematic Reviews and Meta-Analyses
QD	Quantum dot
RCT	Randomized controlled trial
ROI	Region of interest
RUS	Rectal ultrasound
SHAP	Shapley Additive Explanations
SPIRIT-AI	Standard Protocol Items for Interventional Trials with Artificial Intelligence
TAUS	Transabdominal ultrasound
TMB	Tumor mutational burden
TRIPOD-AI	Transparent Reporting of a Multivariable Prediction Model for Individual Prognosis or Diagnosis, Artificial Intelligence Extension
US	Ultrasound
ViT	Vision transformer
WLI	White-light imaging
WSI	Whole-slide image
XAI	Explainable artificial intelligence

## Appendix A. Database-Specific Search Strings

The following database-specific search strings were executed during the identification stage summarized in Figure 1. The declared information sources were PubMed/MEDLINE, Web of Science, IEEE Xplore, and Google Scholar. The search window was January 2018 to August 2026. The searches returned 6017 records from PubMed/MEDLINE, 12,190 from Web of Science, and 899 from IEEE Xplore. Google Scholar was queried as a supplementary source. Where a database interface did not allow the full date range to be encoded directly in the Boolean string, the restriction was applied through the database filters and is reported here.

### Appendix A.1. PubMed/MEDLINE

(
“Colorectal Neoplasms”[MeSH Terms]
OR “Colonic Neoplasms”[MeSH Terms]
OR “Rectal Neoplasms”[MeSH Terms]
OR “Adenomatous Polyps”[MeSH Terms]
OR “Intestinal Polyps”[MeSH Terms]
OR “colorectal cancer”[Title/Abstract]
OR “colorectal cancers”[Title/Abstract]
OR “colorectal neoplasm”[Title/Abstract]
OR “colorectal neoplasms”[Title/Abstract]
OR “colorectal tumor”[Title/Abstract]
OR “colorectal tumour”[Title/Abstract]
OR “colorectal carcinoma”[Title/Abstract]
OR “colorectal adenocarcinoma”[Title/Abstract]
OR “colon cancer”[Title/Abstract]
OR “colonic cancer”[Title/Abstract]
OR “colon carcinoma”[Title/Abstract]
OR “colonic neoplasm”[Title/Abstract]
OR “colonic neoplasms”[Title/Abstract]
OR “rectal cancer”[Title/Abstract]
OR “rectal carcinoma”[Title/Abstract]
OR “rectal neoplasm”[Title/Abstract]
OR “rectal neoplasms”[Title/Abstract]
OR CRC[Title/Abstract]
OR polyp[Title/Abstract]
OR polyps[Title/Abstract]
OR “colorectal polyp”[Title/Abstract]
OR “colorectal polyps”[Title/Abstract]
OR “colon polyp”[Title/Abstract]
OR “colonic polyp”[Title/Abstract]
OR “rectal polyp”[Title/Abstract]
OR adenoma[Title/Abstract]
OR adenomas[Title/Abstract]
OR “colorectal adenoma”[Title/Abstract]
)
AND
(
“Colonoscopy”[MeSH Terms]
OR “Endoscopy, Gastrointestinal”[MeSH Terms]
OR “Colonography, Computed Tomographic”[MeSH Terms]
OR “Endosonography”[MeSH Terms]
OR “Diagnostic Imaging”[MeSH Terms]
OR “Tomography, X-Ray Computed”[MeSH Terms]
OR “Magnetic Resonance Imaging”[MeSH Terms]
OR “Biopsy”[MeSH Terms]
OR “Biomarkers”[MeSH Terms]
OR “Liquid Biopsy”[MeSH Terms]
OR colonoscopy[Title/Abstract]
OR colonoscopic[Title/Abstract]
OR endoscopy[Title/Abstract]
OR endoscopic[Title/Abstract]
OR sigmoidoscopy[Title/Abstract]
OR “CT colonography”[Title/Abstract]
OR “computed tomographic colonography”[Title/Abstract]
OR “virtual colonoscopy”[Title/Abstract]
OR “computed tomography”[Title/Abstract]
OR CT[Title/Abstract]
OR “magnetic resonance imaging”[Title/Abstract]
OR MRI[Title/Abstract]
OR “endoscopic ultrasound”[Title/Abstract]
OR EUS[Title/Abstract]
OR histopathology[Title/Abstract]
OR histopathological[Title/Abstract]
OR pathology[Title/Abstract]
OR “whole slide”[Title/Abstract]
OR “whole-slide”[Title/Abstract]
OR WSI[Title/Abstract]
OR biopsy[Title/Abstract]
OR biopsies[Title/Abstract]
OR biomarker[Title/Abstract]
OR biomarkers[Title/Abstract]
OR “liquid biopsy”[Title/Abstract]
OR “circulating tumor DNA”[Title/Abstract]
OR ctDNA[Title/Abstract]
OR cfDNA[Title/Abstract]
OR “fecal immunochemical test”[Title/Abstract]
OR “faecal immunochemical test”[Title/Abstract]
OR FIT[Title/Abstract]
OR “fecal occult blood”[Title/Abstract]
OR microbiome[Title/Abstract]
OR “multi-omics”[Title/Abstract]
OR metabolomics[Title/Abstract]
)
AND
(
“Artificial Intelligence”[MeSH Terms]
OR “Machine Learning”[MeSH Terms]
OR “Deep Learning”[MeSH Terms]
OR “Neural Networks, Computer”[MeSH Terms]
OR “Diagnosis, Computer-Assisted”[MeSH Terms]
OR “Image Processing, Computer-Assisted”[MeSH Terms]
OR “Natural Language Processing”[MeSH Terms]
OR “artificial intelligence”[Title/Abstract]
OR “machine learning”[Title/Abstract]
OR “deep learning”[Title/Abstract]
OR “neural network”[Title/Abstract]
OR “neural networks”[Title/Abstract]
OR “convolutional neural network”[Title/Abstract]
OR “convolutional neural networks”[Title/Abstract]
OR CNN[Title/Abstract]
OR CNNs[Title/Abstract]
OR transformer[Title/Abstract]
OR transformers[Title/Abstract]
OR “vision transformer”[Title/Abstract]
OR “vision transformers”[Title/Abstract]
OR radiomics[Title/Abstract]
OR radiomic[Title/Abstract]
OR “computational pathology”[Title/Abstract]
OR “digital pathology”[Title/Abstract]
OR segmentation[Title/Abstract]
OR classification[Title/Abstract]
OR “computer-aided detection”[Title/Abstract]
OR “computer-aided diagnosis”[Title/Abstract]
OR “computer-assisted detection”[Title/Abstract]
OR “computer-assisted diagnosis”[Title/Abstract]
OR CADe[Title/Abstract]
OR CADx[Title/Abstract]
OR “multimodal fusion”[Title/Abstract]
OR “large language model”[Title/Abstract]
OR “large language models”[Title/Abstract]
OR LLM[Title/Abstract]
OR “natural language processing”[Title/Abstract]
OR “surgical data science”[Title/Abstract]
OR “foundation model”[Title/Abstract]
OR “foundation models”[Title/Abstract]
)
AND
(
“2018/01/01”[Date - Publication] : “2026/08/31”[Date - Publication]
)
AND “Humans”[MeSH Terms]
AND “English”[Language]
NOT “Preprint”[Publication Type]

### Appendix A.2. Web of Science

TS=(“colorectal cancer*” OR “colorectal neoplasm*” OR “colorectal tumor*” OR “colorectal carcinoma*” OR “colorectal adenocarcinoma*” OR “colon cancer*” OR “colonic neoplasm*” OR “rectal cancer*” OR “rectal neoplasm*” OR “rectal carcinoma*” OR CRC OR polyp* OR “colorectal polyp*” OR “colon polyp*” OR adenoma* OR “colorectal adenoma*”) AND TS=(colonoscop* OR endoscop* OR sigmoidoscop* OR “CT colonography” OR “computed tomographic colonography” OR “virtual colonoscopy” OR “computed tomography” OR CT OR “magnetic resonance imaging” OR MRI OR “endoscopic ultrasound” OR EUS OR histopatholog* OR pathology OR “whole slide*” OR WSI OR biopsy OR biopsies OR biomarker* OR “liquid biopsy” OR “circulating tumor DNA” OR ctDNA OR cfDNA OR “fecal immunochemical test” OR FIT OR “fecal occult blood” OR microbiome OR “multi-omics” OR metabolomics) AND TS=(“artificial intelligence” OR “machine learning” OR “deep learning” OR “neural network*” OR “convolutional neural network*” OR CNN OR transformer* OR “vision transformer*” OR radiomic* OR “computational pathology” OR “digital pathology” OR segmentation OR classification OR “computer-aided detection” OR “computer-aided diagnosis” OR “computer-assisted diagnosis” OR CADe OR CADx OR “multimodal fusion” OR “large language model*” OR LLM OR “natural language processing” OR “surgical data science” OR “foundation model*”)

### Appendix A.3. IEEE Xplore

(“Document Title”:“colorectal” OR “Abstract”:“colorectal cancer” OR “Abstract”:“colon cancer” OR “Abstract”:“rectal cancer” OR “Abstract”:“colorectal neoplasm” OR “Abstract”:“colorectal polyp” OR “Abstract”:“adenoma” OR “Abstract”:“colonoscopy”)
AND
(“Abstract”:“endoscopy” OR “Abstract”:“endoscopic” OR “Abstract”:“colonoscopy” OR “Abstract”:“CT colonography” OR “Abstract”:“computed tomography” OR “Abstract”:“magnetic resonance imaging” OR “Abstract”:“MRI” OR “Abstract”:“endoscopic ultrasound” OR “Abstract”:“histopathology” OR “Abstract”:“pathology” OR “Abstract”:“whole slide” OR “Abstract”:“biopsy” OR “Abstract”:“biomarker” OR “Abstract”:“liquid biopsy” OR “Abstract”:“circulating tumor DNA” OR “Abstract”:“fecal immunochemical test” OR “Abstract”:“microbiome”)
AND
(“Abstract”:“artificial intelligence” OR “Abstract”:“machine learning” OR “Abstract”:“deep learning” OR “Abstract”:“neural network” OR “Abstract”:“CNN” OR “Abstract”:“transformer” OR “Abstract”:“vision transformer” OR “Abstract”:“radiomics” OR “Abstract”:“computational pathology” OR “Abstract”:“digital pathology” OR “Abstract”:“segmentation” OR “Abstract”:“classification” OR “Abstract”:“computer-aided detection” OR “Abstract”:“computer-aided diagnosis” OR “Abstract”:“multimodal fusion” OR “Abstract”:“large language model” OR “Abstract”:“LLM” OR “Abstract”:“natural language processing” OR “Abstract”:“surgical data science”)

Filters: publication years 2018 to 2026.
