# Supplementary material for: Artificial Intelligence for Colorectal Cancer Diagnosis: A Comprehensive Survey of Modalities, Frameworks, and Clinical Tasks
Source: Cancers (Basel). 2026 Nov 2;18(21):3522. doi: 10.3390/cancers18213522 (PMC13762242; doi:10.3390/cancers18213522)
Supplement: Supplementary file 1 [file cancers-18-03522-s001.zip › cancers-4607134-supplementary.pdf]

PRISMA 2020 Checklist

Section and Topic	Item #	Checklist item	Location where item is reported
**TITLE**			
Title	1	Identify the report as a systematic review.	**Title**: "...Comprehensive Survey..."; **Abstract**: "This review synthesizes 160 references...". The 160 figure reflects PRISMA-flow survivors; 7 additional methodological/regulatory citations appear in the bibliography for context only and are not counted in the flow.
**ABSTRACT**			
Abstract	2	See the PRISMA 2020 for Abstracts checklist.	**Abstract:** Page 1.
**INTRODUCTION**			
Rationale	3	Describe the rationale for the review in the context of existing knowledge.	**Section 1 (Introduction)**. **Section 2.5 (Comparison with Existing Surveys):** (identifies three critical gaps in existing literature).
Objectives	4	Provide an explicit statement of the objective(s) or question(s) the review addresses.	**Section 1:** ("Therefore, this review integrates..."). **Section 2.3 (Scope)**.
**METHODS**			
Eligibility criteria	5	Specify the inclusion and exclusion criteria for the review and how studies were grouped for the syntheses.	**Section 2.2**. **Table 1:** Inclusion and exclusion criteria applied during study selection.
Information sources	6	Specify all databases, registers, websites, organisations, reference lists and other sources searched or consulted to identify studies. Specify the date when each source was last searched or consulted.	**Section 2.1**: (explicitly states PubMed/MEDLINE, Web of Science, IEEE Xplore as primary databases; Google Scholar is a supplementary source only and is NOT counted as a primary database). Each of PubMed/MEDLINE, Web of Science, and IEEE Xplore was last searched on 28 August 2026. Figure 1: Lists PubMed/MEDLINE, Web of Science, IEEE Xplore. Appendix A: Database-Specific Search Strings (search window: January 2018 to August 2026; this is explicitly described as a publication-date cutoff applied to the Boolean strings, NOT a search-execution date).
Search strategy	7	Present the full search strategies for all databases, registers and websites, including any filters and limits used.	**Appendix:** "Database-Specific Search Strings" (exact Boolean strings for PubMed/MEDLINE, Web of Science, IEEE Xplore; Google Scholar queried as a supplementary source). Filters: 2018-2026 publication date, English, Humans.
Selection process	8	Specify the methods used to decide whether a study met the inclusion criteria of the review, including how many reviewers screened each record and each report retrieved, whether they worked independently, and if applicable, details of automation tools used in the process.	**Section 2.2:** ("Two reviewers screened records independently at the title and abstract screening stage, achieving a Cohen's kappa of 0.88. Disagreements were resolved by consensus."). The kappa was computed at the title-and-abstract screening stage. **Section 2**: (mentions snowballing for secondary literature).

PRISMA 2020 Checklist

Section and Topic	Item #	Checklist item	Location where item is reported
Data collection process	9	Specify the methods used to collect data from reports, including how many reviewers collected data from each report, whether they worked independently, any processes for obtaining or confirming data from study investigators, and if applicable, details of automation tools used in the process.	**Section 2.2:** ("Two reviewers screened records independently. Disagreements were resolved by consensus. For included studies, reviewers extracted...").
Data items	10a	List and define all outcomes for which data were sought. Specify whether all results that were compatible with each outcome domain in each study were sought (e.g. for all measures, time points, analyses), and if not, the methods used to decide which results to collect.	**Section 2.2:** (extracted performance metrics).<br>**Section 5:** (defines metric conventions: Sensitivity, specificity, AUC, ADR, Dice, IoU).
	10b	List and define all other variables for which data were sought (e.g. participant and intervention characteristics, funding sources). Describe any assumptions made about any missing or unclear information.	**Section 2.2:** (extracted data type, model architecture, clinical task, validation strategy, and principal limitations).
Study risk of bias assessment	11	Specify the methods used to assess risk of bias in the included studies, including details of the tool(s) used, how many reviewers assessed each study and whether they worked independently, and if applicable, details of automation tools used in the process.	**Section 2.4 (Evidence Grading) and Table 2**. PROBAST-AI was not used; the previously listed Supplementary Table S1 has been removed because no PROBAST-AI assessment was performed. A custom structured qualitative evidence-grading framework on three axes (Evidence level, Consistency, Clinical readiness) was applied.<br>**Section 6.1**: discusses analytical bias risks.
Effect measures	12	Specify for each outcome the effect measure(s) (e.g. risk ratio, mean difference) used in the synthesis or presentation of results.	**Section 5:** ("Metric definitions: Sensitivity and specificity refer to per-lesion or per-patient performance... AUC refers to the area under the receiver operating characteristic curve...").
Synthesis methods	13a	Describe the processes used to decide which studies were eligible for each synthesis (e.g. tabulating the study intervention characteristics and comparing against the planned groups for each synthesis (item #5)).	**Section 5:** ("Within-task comparison only: Metrics are compared only among studies addressing the same clinical task on the same modality.").
	13b	Describe any methods required to prepare the data for presentation or synthesis, such as handling of missing summary statistics, or data conversions.	**Section 5:** ("No pooled estimation without meta-analysis... Narrative synthesis is used instead.").
	13c	Describe any methods used to tabulate or visually display results of individual studies and syntheses.	**Tables 13–19:** Summarize individual studies by clinical task.<br>**Figure 1:** PRISMA flow diagram.
	13d	Describe any methods used to synthesize results and provide a rationale for the choice(s). If meta-analysis was performed, describe the model(s), method(s) to identify the presence and extent of statistical heterogeneity, and software package(s) used.	**Section 2.4:** ("Because included studies report heterogeneous metrics, validation designs, and population characteristics, formal meta-analysis was not feasible... structured qualitative evidence grading was applied").
	13e	Describe any methods used to explore possible causes of heterogeneity among study results (e.g. subgroup analysis, meta-regression).	**Not Applicable.** Formal meta-analysis was not performed due to heterogeneity (Section 2.4).
	13f	Describe any sensitivity analyses conducted to assess robustness of the synthesized results.	**Not Applicable.** No meta-analysis was performed.
Reporting bias assessment	14	Describe any methods used to assess risk of bias due to missing results in a synthesis (arising from reporting biases).	**Section 6.1:** (Addresses analytical bias, patient-level overlap, and benchmark contamination that can inflate reported performance).

PRISMA

PRISMA 2020 Checklist

Section and Topic	Item #	Checklist item	Location where item is reported
Certainty assessment	15	Describe any methods used to assess certainty (or confidence) in the body of evidence for an outcome.	**Section 2.4** and **Table 2** (Assesses Evidence Level, Consistency, and Clinical Readiness for each clinical task).
**RESULTS**			
Study selection	16a	Describe the results of the search and selection process, from the number of records identified in the search to the number of studies included in the review, ideally using a flow diagram.	**Section 2**.<br>**Figure 1:** PRISMA 2020 style literature selection flow diagram.
	16b	Cite studies that might appear to meet the inclusion criteria, but which were excluded, and explain why they were excluded.	**Figure 1:** Details specific reasons and counts for full-text exclusions (Out of scope n=124; No primary AI model development n=100; Non-human/preclinical n=80; Purely narrative commentaries n=40; Insufficient detail n=55; Overlapping/duplicate n=35; total n=434).
Study characteristics	17	Cite each included study and present its characteristics.	**Tables 13–19:** Summarize characteristics (Study, Architecture/Modality, Clinical Task, Best Performance, Principal Limitation).<br>**References**.
Risk of bias in studies	18	Present assessments of risk of bias for each included study.	**Tables 13–19:** “Principal Limitation” column for each study.<br>**Section 6.1:** (Discusses overarching risks like patient-level overlap and benchmark contamination).
Results of individual studies	19	For all outcomes, present, for each study: (a) summary statistics for each group (where appropriate) and (b) an effect estimate and its precision (e.g. confidence/credible interval), ideally using structured tables or plots.	**Tables 13–19:** “Best Performance” column details specific metrics (e.g., ADR, AUC, Sensitivity, Specificity, Dice) for each study.
Results of syntheses	20a	For each synthesis, briefly summarise the characteristics and risk of bias among contributing studies.	**Section 5 (Subsections 5.1–5.7):** Narrative summaries of characteristics and limitations for each specific clinical task.
	20b	Present results of all statistical syntheses conducted. If meta-analysis was done, present for each the summary estimate and its precision (e.g. confidence/credible interval) and measures of statistical heterogeneity. If comparing groups, describe the direction of the effect.	**Not Applicable.** Meta-analysis was not feasible (Section 2.4). Narrative synthesis is provided in Section 5.
	20c	Present results of all investigations of possible causes of heterogeneity among study results.	**Not Applicable.** (No meta-analysis performed). Heterogeneity is discussed qualitatively in Section 2.4 and 6.1.
	20d	Present results of all sensitivity analyses conducted to assess the robustness of the synthesized results.	**Not Applicable.** (No meta-analysis performed).
Reporting biases	21	Present assessments of risk of bias due to missing results (arising from reporting biases) for each synthesis assessed.	**Section 6.1**. The previously listed “publication bias assessment in Section 7” has been removed because no such assessment was performed.
Certainty of evidence	22	Present assessments of certainty (or confidence) in the body of evidence for each outcome assessed.	**Table 2:** Structured evidence grading by clinical task.<br>**Section 7:** (Categorizes evidence maturity as “Established”, “Validated but Not Yet Deployed”, or “Exploratory”).
**DISCUSSION**			

PRISMA 2020 Checklist

Section and Topic	Item #	Checklist item	Location where item is reported
Discussion	23a	Provide a general interpretation of the results in the context of other evidence.	**Section 7:** Open Challenges and Future Directions.<br>**Section 8:** Conclusions.
	23b	Discuss any limitations of the evidence included in the review.	**Section 6.1**.<br>**Section 7:** Cross-Cutting Barriers (e.g., domain shift, limited prospective evidence).
	23c	Discuss any limitations of the review processes used.	**Section 2.4:** (Formal meta-analysis not feasible due to heterogeneity).<br>**Section 6.1:** (Splitting at the patient level was not explicitly reported in most included studies).
	23d	Discuss implications of the results for practice, policy, and future research.	**Section 7:** Future Work, Clinical Implementation Pathway, and Regulatory Pathways.<br>**Section 8:** Conclusions.
**OTHER INFORMATION**			
Registration and protocol	24a	Provide registration information for the review, including register name and registration number, or state that the review was not registered.	**Not registered in PROSPERO (stated explicitly in Section 2.1).**
	24b	Indicate where the review protocol can be accessed, or state that a protocol was not prepared.	**No protocol prepared (stated explicitly in Section 2.1).**
	24c	Describe and explain any amendments to information provided at registration or in the protocol.	**Not applicable.**
Support	25	Describe sources of financial or non-financial support for the review, and the role of the funders or sponsors in the review.	**Funding Statement:** (“This research received no external funding.”)
Competing interests	26	Declare any competing interests of review authors.	**Conflicts of Interest:** (“The authors declare no conflicts of interest.”)
Availability of data, code and other materials	27	Report which of the following are publicly available and where they can be found: template data collection forms; data extracted from included studies; data used for all analyses; analytic code; any other materials used in the review.	**Data Availability Statement:** No new data were created or analyzed in this study. The extracted dataset of 160 included studies, along with the list of full-text exclusions and the PRISMA 2020 checklist, is provided in the Supplementary Materials.

From: Page MJ, McKenzie JE, Bossuyt PM, Boutron I, Hoffmann TC, Mulrow CD, et al. The PRISMA 2020 statement: an updated guideline for reporting systematic reviews. BMJ 2021;372:n71. doi: 10.1136/bmj.n71. This work is licensed under CC BY 4.0. To view a copy of this license, visit https://creativecommons.org/licenses/by/4.0/
